# Modulating gut microbiota in a mouse model of Graves’ orbitopathy and its impact on induced disease

**DOI:** 10.1186/s40168-020-00952-4

**Published:** 2021-02-16

**Authors:** Sajad Moshkelgosha, Hedda Luise Verhasselt, Giulia Masetti, Danila Covelli, Filippo Biscarini, Mareike Horstmann, Anke Daser, Astrid M. Westendorf, Christoph Jesenek, Svenja Philipp, Salvador Diaz-Cano, J. Paul Banga, Daryn Michael, Sue Plummer, Julian R. Marchesi, Anja Eckstein, Marian Ludgate, Utta Berchner-Pfannschmidt, and the INDIGO consortium

**Affiliations:** 1 Molecular Ophthalmology, Department of Ophthalmology, University Hospital Essen, University of Duisburg-Essen, 45147 Essen, Germany; 2 Current address: Latner Thoracic Surgery Laboratories, Toronto General Research Institute, University Health Network and University of Toronto, Toronto, Canada; 3 Institute of Medical Microbiology, University Hospital Essen, University of Duisburg-Essen, Essen, Germany; 4 Cultech Ltd., Baglan, Port Talbot, UK; 5 Division of Infection & Immunity, School of Medicine, Cardiff University, UHW main building, Heath Park, Cardiff, CF14 4XW UK; 6 Department of Bioinformatics, PTP Science Park Srl, Lodi, Italy; 7 Current address: Computational metagenomics, Department CIBIO, University of Trento, Trento, Italy; 8 Graves’ Orbitopathy Center, Endocrinology, Department of Clinical Sciences and Community Health, Fondazione Ca’Granda IRCCS, University of Milan, Milan, Italy; 9 Italian National Research Council (CNR), Milano, Italy; 10 Department of Ophthalmology, University Hospital Essen, University of Duisburg-Essen, 45147 Essen, Germany; 11 Department of Histopathology, King’s College Hospital, King’s College, London, UK; 12 School of Biosciences, Cardiff University, Cardiff, UK; 13 Department of Metabolism, Digestion and Reproduction, Imperial College London, London, UK

**Keywords:** Graves’ disease, Graves’ orbitopathy, Murine model, Gut microbiota, Microbiome modulation, Vancomycin, Probiotics, Human fecal microbiota transplant

## Abstract

**Background:**

Graves’ disease (GD) is an autoimmune condition in which autoantibodies to the thyrotropin receptor (TSHR) cause hyperthyroidism. About 50% of GD patients also have Graves’ orbitopathy (GO), an intractable disease in which expansion of the orbital contents causes diplopia, proptosis and even blindness. Murine models of GD/GO, developed in different centres, demonstrated significant variation in gut microbiota composition which correlated with TSHR-induced disease heterogeneity. To investigate whether correlation indicates causation, we modified the gut microbiota to determine whether it has a role in thyroid autoimmunity. Female BALB/c mice were treated with either vancomycin, probiotic bacteria, human fecal material transfer (hFMT) from patients with severe GO or ddH2O from birth to immunization with TSHR-A subunit or beta-galactosidase (βgal; age ~ 6 weeks). Incidence and severity of GD (TSHR autoantibodies, thyroid histology, thyroxine level) and GO (orbital fat and muscle histology), lymphocyte phenotype, cytokine profile and gut microbiota were analysed at sacrifice (~ 22 weeks).

**Results:**

In ddH2O-TSHR mice, 84% had pathological autoantibodies, 67% elevated thyroxine, 77% hyperplastic thyroids and 70% orbital pathology. *Firmicutes* were increased, and *Bacteroidetes* reduced relative to ddH2O-βgal; CCL5 was increased. The random forest algorithm at the genus level predicted vancomycin treatment with 100% accuracy but 74% and 70% for hFMT and probiotic, respectively. Vancomycin significantly reduced gut microbiota richness and diversity compared with all other groups; the incidence and severity of both GD and GO also decreased; reduced orbital pathology correlated positively with *Akkermansia* spp. whilst IL-4 levels increased. Mice receiving hFMT initially inherited their GO donors’ microbiota, and the severity of induced GD increased, as did the orbital brown adipose tissue volume in TSHR mice. Furthermore, genus *Bacteroides*, which is reduced in GD patients, was significantly increased by vancomycin but reduced in hFMT-treated mice. Probiotic treatment significantly increased CD25^+^ Treg cells in orbital draining lymph nodes but exacerbated induced autoimmune hyperthyroidism and GO.

**Conclusions:**

These results strongly support a role for the gut microbiota in TSHR-induced disease. Whilst changes to the gut microbiota have a profound effect on quantifiable GD endocrine and immune factors, the impact on GO cellular changes is more nuanced. The findings have translational potential for novel, improved treatments.

Video abstract

**Supplementary Information:**

The online version contains supplementary material available at 10.1186/s40168-020-00952-4.

## Background

There is an interdependent relationship between the host immune system and the gut microbiota. Differentiation of naïve CD4^+^ T cells in the gut-associated lymphoid tissue is modulated by microbial metabolites [1]. Consequently, autoimmune disease, including type-1 diabetes (T1D) and multiple sclerosis (MS), is influenced by the gut microbiota composition [2–7]. Perturbation of the gut microbiota may improve or exacerbate autoimmunity, for example, administration of antibiotic to female systemic lupus erythematosus-prone mice attenuates disease [8], but human fecal material transfer (hFMT) from MS patients increased the frequency of murine autoimmune encephalomyelitis (EAE) [9]. In contrast, administration of probiotics to EAE mice resulted in a milder disease phenotype [10] and reduced inflammation in a model of autoimmune thyroid disease (ATD) [11].

Little is known regarding the role of the microbiome in Graves’ disease (GD), which is characterized by thyroid-stimulating antibodies (TSAbs) binding to the thyrotropin (TSH) receptor (TSHR) resulting in thyroid hyperplasia and hyperthyroidism [12–14]. The TSHR is expressed in orbital fibroblasts and up to 50% of GD patients also have Graves’ orbitopathy (GO), in which orbital tissue remodeling causes eye redness, swelling, diplopia, proptosis and vision impairment [15–17]. Genetic background and environmental factors, e.g. stress, smoking and pregnancy, combine to increase predisposition to GD and GO [18–22]. Bacterial infections have also been implicated [23], as have immune responses to food and other antigens [24].

We recently described a relationship between the gut microbiota and thyroid autoimmunity [25] in a robust GO mouse model in which female BALB/c (H-2d) mice were immunized with the TSHR-A subunit (TSHR extracellular region) via plasmid electroporation [26, 27]. Differences in the induced disease were noted in the same mouse strain (BALB/c) but in two different laboratories, e.g. hyperthyroidism only in one centre accompanied by significant differences in alpha-diversity, beta-diversity and taxonomic profiles between the two centres. We also observed a shift in beta-diversity of bacterial communities in TSHR-immunized mice compared to controls and a positive correlation between Firmicutes and orbital adipogenesis [25, 28]. When comparing mice of differing strains (BALB/c and C57BL/6J), but in the same housing, we observed variations in orbital pathology, TSAbs, thyroxine levels and inflammation, accompanied by differences in microbiome composition. Bacterial genera were also correlated with disease features, e.g. *Clostridium*-IV and *Anaerotruncus* spp. positively correlated with TSAb levels in BALB/c mice whereas *Limibacter* spp. correlated negatively with TSAbs in C57BL/6J mice [25, 28].

However, correlation does not imply causation, and to test the microbiota contribution to the GO model from early life, we modulated its composition and assessed induced disease phenotype.

## Materials and methods

### Mouse model and study outline

**Fig. 1:**
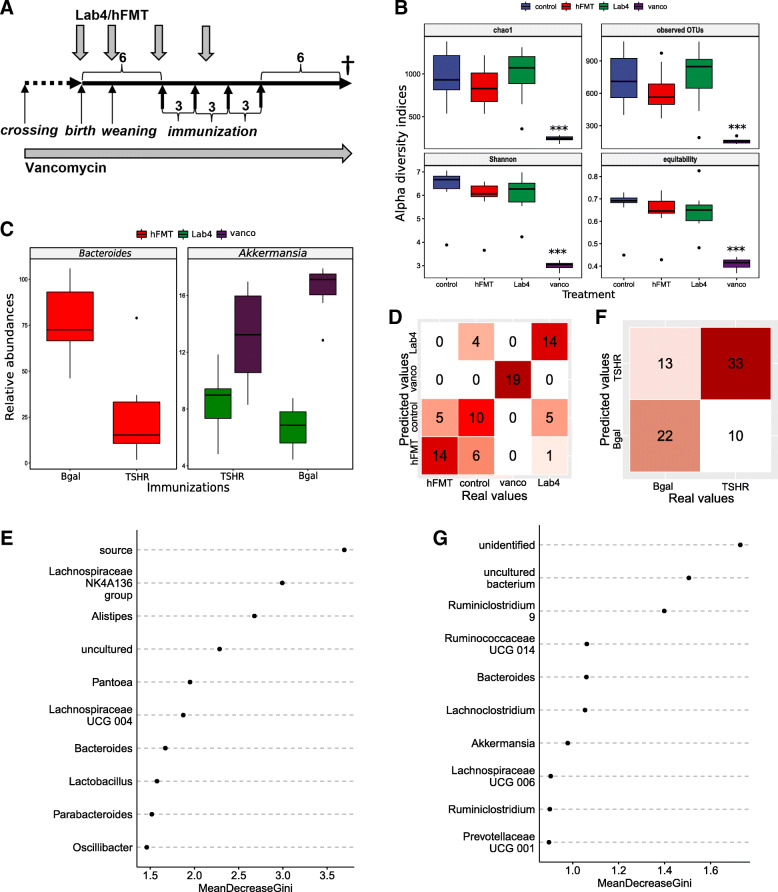
Early-life manipulation treatments modified the lower gastrointestinal microbiota with long-term effects in TSHR-immunized mice. **a** Rationale of early-life manipulation treatments combined with the hTSHR-immunization procedure, see STAR methods for complete description. **b** Alpha-diversity indices amongst treatments. Wilcoxon-Mann test BH corrected: ***P < 0.001; **P = 0.019; *P = 0.045. **c** Significantly differentially abundant genera between immunizations in each treatment. Welch’s *t* test, BH corrected: ***P < 0.001; **P = 0.011; *P = 0.04. **d** Random forest confusion matrix of the classification for treatments (all immunizations together). Diagonal boxes represent the number of samples correctly predicted. The RF model accounted for 73.08% accuracy (*n* mice/group endpoint: control = 20, hFMT = 19, Lab4 = 20, vancomycin = 19). **e** Top-10 important variables contributing to treatment classification according to the Mean Decrease Gini, related to **e**. **f** RandomForest confusion matrix of the classification for immunizations. The RF model accounted for 70.51% accuracy (*n* mice/immunization endpoint: TSHR = 43, βgal = 35). **g** Top-10 important variables contributing to immunization classification according to the Mean Decrease Gini, related to **g**

Female BALB/c mice used in this study were bred at the Central Animal Laboratory, University Hospital Essen, University of Duisburg-Essen, Essen, in order to administer the treatments from an early stage of life. Breeding pairs were purchased from Harlan, now Envigo, Rossdorf, Germany. In total, 95 mice were included in the study. Three of the 95 mice died for unknown reasons. The mouse cohort was divided into four groups receiving either (i) the antibiotic vancomycin, (ii) the probiotic consortium Lab4, (iii) freeze-dried human fecal material from severely diseased GO patients and (iv) deionized water (ddH_2_O) as control. The antibiotic vancomycin was provided in autoclaved drinking water at a dose of 0.2 g/l to both dams first and pups later from their first day of life for the entire course of the experiment. The probiotic Lab4 (Cultech Ltd., Port Talbot, Wales, UK) is a consortium comprising two strains of *Lactobacillus acidophilus* CUL60 (NCIMB 30157) and CUL21 (NCIMB 30156), *Bifidobacterium lactis* CUL34 (NCIMB 30172) and *Bifidobacterium bifidum* CUL20 (NCIMB 30153). It was provided as powder in a capsule, and loose powder was administered at a total of 1 × 10^10^ CFU in 50 μl autoclaved water per gavage. The hFMT powder was dissolved in autoclaved water and provided at a final concentration of 1 × 10^10^ CFU in 50 μl autoclaved water per gavage. Administration of the probiotic Lab4, the hFMT powder and ddH_2_O as control was performed through gavage on pups a total of four times from the first day after birth, at weaning, and before and in the mid of the immunization procedure, as described in Fig. 1a.

At an age of 6–7 weeks and after having received three gavages or vancomycin continuously, mice from each group were assigned to two subgroups for receiving immunization with either the human thyrotropin receptor (hTSHR)-A subunit (hTSHR) eukaryotic expression plasmid pTriEx1.1Neo-hTSHR (hTSHR289) or the control plasmid pTriEx1.1Neo-β-gal, as a plasmid-control group (βgal) via intramuscular injection with 50 μl (1 mg/ml) into each M. biceps femoris and electroporation four times at three week intervals [26, 29, 30]. All plasmids were purified from *E. coli* extracts and stored aliquoted at − 80 °C. As an electroporation system, a BTX Gemini X2 with 7-mm calliper electrodes at 120 V/cm was used with current application in two series of six 20-msec^2^ wave pulses at 1 Hz. Anaesthesia of each mouse was conducted with an isoflurane vaporizer throughout the immunization procedure. Mice were kept in individually ventilated cages under specific pathogen-free (SPF) conditions throughout the study. Animals were fed water and commercial diet ad libitum. Animals were sacrificed 6 weeks after the last immunization or at an age of 22 weeks, respectively. Mouse weights were noted at the endpoint of the study (balance 440-45N (Kern und Sohn GmbH, Balingen, Germany).

### Recruitment of patients with severe Graves’ orbitopathy for human fecal microbiota transplant (hFMT) production

Six Graves’ orbitopathy patients with sight-threatening disease were recruited at the Ophthalmic Clinic of the University Hospital of Duisburg-Essen (Germany). Eye disease activity and severity were assessed based on the EUGOGO guidelines [31]. Five of the six patients stopped steroid intake at least 2 months prior to sample collection, whereas one patient took steroids in the month of sampling. None of the six patients received immunosuppressive drugs before sampling, and receipt of antibiotics in the previous 3 months excluded potential donors. All six patients were treated with steroid bolus and selenium before orbital decompression surgery (performed between 2014 and 2015). One patient (GO 4) had the decompression of both eyes, and two patients (GO 4 and GO 5) continued steroid treatment after surgery. Assessment of thyroid blood parameters (TSH and total serum T4) and thyroid-stimulating antibodies (TRAB) was performed according to bioassays used in the central laboratory at University Hospital Essen, Germany. A complete description of the patient characteristics used for hFMT production is described in Table S1. Donors were instructed in person before the procedure regarding stool collection and delivery. Subsequent fecal samples were collected at the time of enrolment, stored at − 80 °C and shipped frozen to Cultech Ltd. (Port Talbot, UK) for the production of the fecal microbiota transplant (hFMT). Each single fecal sample underwent traditional microbial cultures and DNA extraction for 16S rRNA gene sequencing.

### Production of freeze-dried fecal bacteria for human fecal microbiota transplant (hFMT)

To assess numbers of living bacteria in feces, 1 gram of feces per patient was diluted in 9 ml pre-reduced maximum recovery diluent (CM0733, Oxoid, Basingstoke, UK) with 20% v/v glycerol. The solution was mixed by vortexing for 5 s. Then, 10-fold serial dilutions were prepared, and 100 μl of each dilution was plated onto different culture media under aerobic or anaerobic conditions (Anaerobic Workstation, AW400SG, Elektrotek, Keighley, West Yorkshire, UK). In order to isolate different bacteria, the following media, culture conditions and dilutions were used as described in [25], see additional methods. For hFMT production, blended fecal samples were run through a sequential culture method. Therefore, 0.1 g of pooled and blended feces was added to 50 mL pre-reduced maximum recovery diluent (MRD) broth and incubated overnight at 37 °C under aerobic and anaerobic conditions. The mixture was further inoculated into 500 mL pre-reduced MRD, followed by an overnight incubation at 37 °C under aerobic and anaerobic conditions. As a control, pooled fecal samples from each inoculum were plated on the non-selective agars horse blood agar and anaerobic blood agar (both purchased from Thermo Fisher Scientific Oxoid Ltd, Basingstoke, UK) and incubated overnight at 37 °C under aerobic or anaerobic conditions in order to count viable cells. After a centrifugation step at 3000×*g* for 30 min, the resulting supernatant was discarded and the pellet was weighed and poured into sterile petri dishes, where they were supplemented with 10% w/v skimmed milk powder as a cryoprotectant agent, and placed at − 80 °C until completely frozen. The freeze-drying process was performed in a freeze-dryer machine (Super Modulyo freeze dryer, Edwards, Clevedon, North Somerset, UK). The frozen sample was moved into the freeze-drier with a shelf temperature of − 30 °C; afterwards, the vacuum was started, the temperature was slowly decreased to − 15 °C and the samples were kept under these conditions overnight. Finally, the temperature was raised to 0 for 2 h and subsequently raised to 20 °C for another 2 h. To count viable bacterial cells in the hFMT powder for quality purposes, 50 μl from a stock of 0.5 g hFMT powder was diluted in 4.5 mL MRD, streaked out on non-selective agars (horse blood agar for total aerobic bacteria and anaerobic blood agar for total anaerobe bacteria) as well as on agars selective for lactobacilli (Man, Rogosa and Sharpe broth (MRS) agar) and for bifidobacteria (MRSx agar). Agars were incubated overnight at 37 °C or for 48 h in the case of MRSx agar; conditions were aerobic (horse blood agar and anaerobic blood agar) or anaerobic conditions (MRS and MRSx), respectively. The resulting powder was aliquoted into small vials to 0.125 g final content and shipped to the University Hospital Essen.

### Mouse fecal sample collection

Individual fecal pellets from mice were collected after three treatment gavages but before any immunizations with hTSHR or βgal (baseline), and after four gavages but before the third immunization (mid-timepoint). The contents of colon or entire intestines were collected for metataxonomic analysis after the sacrifice of the mice (6 weeks after the last immunization and nearly 9 weeks after the last gavage).

### DNA extraction and sequencing

DNA was extracted using the QiAmp Fast DNA Stool Mini kit (Qiagen Ltd, West Sussex, UK). Collected samples were individually placed in 2-mL tubes prefilled with 0.1 mm silica and zirconia bead mix (Benchmark Scientific, Edison, USA), dissolved in 1 mL InhibitEX buffer (Qiagen Ltd, West Sussex, UK) and vortexed until homogenized. A bead-beating step (Beadbug microcentrifuge homogenizer, Benchmark Scientific, USA) was applied for 3 x 60 s at 5 m/s with 5-min rest in between. Total genomic DNA was eluted in sterile microcentrifuge tubes and quantified by Qubit Fluorimetric Quantitation (ThermoFisher Scientific Ltd, UK) as per manufacturer’s instructions. Metataxonomic sequencing (16S rRNA gene sequencing) was performed at Research & Testing RTL Genomics (Lubbock, TX, USA), using primers detecting the V1-V2 regions of the 16S rRNA gene plus bifidobacteria regions to generate 10,000 paired-ends reads on a Illumina MiSeq (Illumina, San Diego, USA.

### Processing of sequencing reads

A first quality check on raw demultiplexed paired-end sequences (R1 and R2) was done using FastQC. All of the below steps were performed with the quantitative insights into microbial ecology (QIIME) 1.9 open-source bioinformatics pipeline for microbiome analysis [32]. Joining of paired-end sequences was done using the function “multiple_join_paired_end.py”, with the SeqPrep method (https://github.com/jstjohn/SeqPrep). Quality filtering of the reads was performed according to the following parameters: (i) maximum of three consecutive low-quality base calls (Phred < 19) allowed and (ii) fraction of consecutive high-quality base calls (Phred > 19) in a read over total read length > = 0.75; iii) no “N”-labeled bases (missing/uncalled) allowed. A total of 13,782,107 reads were obtained before quality filtering including also the small intestines samples. The reads per group were as the following: ddH_2_O 2,593,620; hFMT 2,972,296; Lab4 2,102,509; vancomycin 5,280,854 and GO patients 483,510 (*n* = 6, i.e. reads were sequenced in a separate run with the same primers and were processed together with mouse reads processing using QIIME and unknown 349,318). Reads were reduced to a total of 12,884,785 after quality filtering (reads per group: ddH_2_O 2,418,786; hFMT 2,757,051; Lab4 1,945,969; vancomycin 5,003,546; GO patients 428,852 and unknown 330,581). Passing-filter reads were aligned against the SILVA 123 reference database using the “pick_closed_reference_otus.py” approach for taxonomic assignment with a 97% cluster identity [33]. Operational taxonomic unit (OTUs) with total counts lower than 15 in fewer than 2 samples were filtered out. To correct potential biases in library size due to sampling procedures or sequencing depth, OTUs were normalized in each library through the cumulative sum scaling (CSS) [34] implemented in the “normalized_table.py” function. Filtered and normalized OTUs were collapsed into each phylogenetic level (from phylum to genus) using the function “taxa_summary.py”.

### Serological analysis of mouse blood

Anti-TSHR antibodies (TRAbs) and TSH-stimulating antibodies (TSAbs) and total T4 were evaluated in mouse serum [27, 35]. Therefore, TRAbs were measured in commercial TRAK kits using 25 μl serum plus 75 μl human control serum as TSH binding inhibitory immunoglobulins activity in competition with labeled bTSH to the human TSHR (ThermoFisher, BRAHMS, Germany). TSAbs were measured in 3 μl serum plus 147 μl buffer in a bioassay using stably transfected mouse TSHR-Chinese hamster ovary cells (CHO) cells kindly provided by Sandra McLachlan [29, 30]. TSHR-CHO cells were incubated with test sera diluted 1:20 in Ham’s F12 supplemented with HEPES 10 mM (pH 7.4) and isobutylmethylxanthine 1 mM in 96-well plates for 80 min at 37 °C. Intracellular cAMP was extracted with ethanol after aspirating the medium, evaporated to dryness and resuspended in Dulbecco’s PBS. The concentration of TSAbs is directly correlated to the cAMP production of the cells. cAMP concentration in 100 μl of the mouse TSHR-CHO cells supernatants was measured by ELISA (Enzo, Farmingdale, NY, USA). Total T4 concentrations were measured in 25 μl serum by ELISA (DRG, Springfield, NJ, USA).

### Histopathology of thyroid glands and orbits

The thyroids were formalin fixed and paraffin embedded, and sections (1 μm) H&E stained. Thyroidal morphology was blindly evaluated by two different observers and indexed as hyperactive, heterogeneous or normal in comparison to the thyroid morphology of the respective βgal mice. Hyperplastic glands (indicating hyperthyroidism) were characterized by increased total size, cuboid cylindrical follicular cells with minimal colloid and thick follicular epithelium. Numbers of mice displaying normal, heterogeneous or hyperactive thyroid morphology are given in %. The orbits were formalin fixed and paraffin embedded. Orbital slices (1 μm) at the anterior, middle and posterior area of the mouse orbits were H&E stained and examined. Quantification of adipose tissues and muscle atrophy in extraocular inferior rectus and medial rectus muscle was performed with ImageJ. The area of adipose tissue was normalized to the optic nerve area. Atrophic cells were identified by diameter (< 50 μm) and round shape of muscle fibers [27, 35]. Images were generated using an Olympus BX51 microscope.

### Analysis of lymph node T cell subsets by flow cytometry

Draining lymph nodes of mouse orbits were harvested and teased apart into a single-cell suspension by mashing tissue between two frosted microscope slides. Collected cells were filtered using a 100-μm cell strainer to eliminate clumps and before centrifuging 5 min 400×*g* at 4 °C. Cell pellet was then resuspended in 1 ml of PBS with 2% FBS to count and evaluate cell viability. Appropriate volumes of each sample equal to 2 × 10^6^ cells were transferred to new tubes for staining. Antibody mix of CD4 FITC, CD8PE and CD25 APC (all from BioLegend) was added to each sample and added up to 100 μl using PBS with 2% FBS. Cells were incubated for at least 30 min at 2–8 °C before washing and spinning down at 400×*g* for 5 min. Cells were resuspended in 300 μL of PBS with 2% FBS and subjected to read by flow cytometry.

### Measurement of circulating cytokines in mouse serum

CCL5, IL-4, CCL20, IFN-γ, IL-2, IL-6, IL-10, IL-17/IL-17A and TNF-alpha in murine sera were measured by polystyrene bead-based Luminex technology (R&D Systems, Minneapolis, USA) according to the manufacturer’s instructions, and the assay was run on a Luminex 200 instrument (Luminex Corporation, Austin, TX, USA).

### Statistical analysis

Results are presented as mean ± standard error of the mean. Multiplicity adjusted *P* values are marked as follows: *≤ 0.05; **≤ 0.01; ***≤ 0.001; ****≤ 0.0001. Changes between mouse groups with *P* values > 0.05 are regarded as not statistically significant and are not marked in the graphs. Additionally, the upper 99% confidence interval (CI) of the control βgal group was defined as the threshold for positivity of individual mice and is indicated by a dotted line when appropriate. In order to compare the total outcome between the groups, we combined the key parameters of either autoimmune hyperthyroidism (TSAbs and T4) or orbitopathy (brown adipose tissue (BAT) and atrophic fibers) by using the Z-score method. The standard score (Z-score) was used to compare results from different mouse groups normalized to the mean value of the total mouse population (reference population). The Z-score values (arbitrary units) represent the values of standard deviation from the mean value of the reference population. We also categorized severity of induced disease: Subclinical disease (Z-score < 0): These mice did not develop significant autoimmune hyperthyroidism and/or GO. Clinical disease (Z-score > 0): These mice displayed signs of overt disease during the experiment. Clinical disease is classified in accordance with Z-score values as mild (Z-score 0 > 1) or moderate-to-severe (Z-score > 1). The number of mice is given in %. Bacterial counts data were Box-Cox transformed before statistical analysis [36]. Otherwise stated, statistical analyses for 16S rRNA gene sequencing data were performed in R v3.4.1. Alpha- and beta-diversity indices were obtained from the filtered OTU-table in QIIME. Kruskal-Wallis and pairwise Wilcox-test with Benjamini-Hochberg (BH) adjustment for multiple corrections were used to test the association between alpha-diversity indices and variables (e.g. immunizations, treatments or microbiota sources). Dissimilarities amongst groups (treatments/immunizations) or pairwise dissimilarities along variables were evaluated non-parametrically using the permutational analysis of variance approach (PERMANOVA) with 999 permutations [37], implemented in the R Vegan package. When necessary, a stratification of the permutations was applied to correct for the different microbiota sources sampled (e.g. entire and colon samples). Differential abundant taxonomies were identified using a linear regression model. Random forest (RF) was employed to classify samples either amongst treatments (ddH_2_O, hFMT, Lab4 or vancomycin) or between immunizations (βgal or TSHR) based on their microbiota composition and to identify genera driving the classification (variable importance). Correlations between disease features and bacterial biomarkers were assessed using the Pearson’s correlation coefficient (*r*), using the Corrplot R package. The extent of the hFMT between donors and recipients—or engraftment [38]—was calculated using the SourceTracker R package [39]. A detailed description of the methods performed is available in Additional Methods.

## Results

The mouse cohort comprised four groups receiving different treatments: (i) vancomycin antibiotic; (ii) Lab4 probiotic consortium (2 strains of *Lactobacillus acidophillus*, 1 strain *Bifidobacterium animalis*, 1 strain *Bifidobacterium bifidum*); (iii) freeze-dried human fecal material (hFMT) from 6 severe GO patients; (iv) ddH2O as control, as described in Fig. 1a. Pups were gavaged with either Lab4 or hFMT or ddH_2_O 1 day after birth, at weaning, and before and in the middle of immunizations whereas dams and their pups (from birth) received vancomycin in their drinking water throughout the experiment.

### Early-life manipulation treatments conferred enduring changes of the TSHR-immune lower gastrointestinal microbiota

At the end of the experimental procedure (Fig. 1a), 9 weeks after the last gavaged treatment, the microbiota was sampled and analysed via 16S rRNA gene sequencing, from the large intestines in vancomycin, hFMT and ddH_2_O groups. The gut microbiota was obtained from the entire intestine of Lab4-treated and a related group of ddH_2_O-receiving mice. A total of 3623 OTUs with more than 15 counts in at least two samples were retrieved from quality-filtered reads and used in further analyses. There were no significant differences in alpha- and beta-diversity indices between the entire and the large intestines (Figure S1A–B); therefore, data from the two anatomical sources were combined and referred to as lower gastrointestinal tract (LGI) microbiota.

Vancomycin-treated LGI microbiota had drastically reduced richness and diversity compared to ddH_2_O, hFMT and Lab4 mice, in both TSHR and βgal-immunized mice (Fig. 1b and S1C, respectively). Firmicutes-prevalent genera such as *Faecalibacterium*, *Eubacterium, Ruminiclostridium* and *Ruminococcaceae* spp. were depleted from vancomycin LGI microbiota in both TSHR (Figure S2) and βgal mice (Figure S3), whilst *Bacteroides* spp. showed the highest counts compared to ddH_2_O-, Lab4 -and hFMT-TSHR-immunized mice. Potential vancomycin-resistant bacteria were present, including Proteobacteria genera *Enterobacter*, *Salmonella*, *Pseudomonas* spp. and *E. coli*. Interestingly, Verrucomicrobia genus *Akkermansia* increased in TSHR compared to βgal vancomycin-treated mice (*P* = 0.011, Fig. 1c). The unique vancomycin microbiota composition facilitated 100% per-class prediction accuracy (19/19) using the random forest algorithm with 10,000 decision trees, to predict early-life treatments in endpoint samples’ genus-level gut microbiota (Fig. 1d–g).

TSHR-Lab4 mice showed a lower equitability index compared to TSHR-ddH_2_O mice (*P* = 0.045, Fig. 1b). Differentially abundant genera were observed after Lab4 administration, with twelve genera showing increased or decreased relative abundance when compared with TSHR-ddH_2_O mice (data not shown). In contrast to the vancomycin treated mice, *Akkermansia* spp. decreased in TSHR-Lab4 compared to βgal-Lab4 (*P* = 0.040, Fig. 1c).

Bacteria in the hFMT powder were viable (Figure S4A). TSHR-hFMT had reduced Shannon diversity compared to TSHR-ddH_2_O mice (*P* = 0.019, Fig. 1b), whilst no significant differences occurred in βgal mice (Figure S1A). At genus level, TSHR-hFMT mice showed significantly lower *Bacteroides* spp. counts compared to ddH_2_O (*P* = 0.003, Figure S2). Following pairwise comparison, sixteen genera were differentially abundant compared to the TSHR-ddH_2_O group (data not shown). *Bacteroides* spp. were significantly reduced in hFMT-TSHR compared to hFMT-βgal mice (*P* < 0.001, Fig. 1c), as previously reported [25] and between GO patients and healthy controls (Figure S4B–D).

Differences in the Lab4 and hFMT LGI microbiota composition were sufficient for predicting treatment with per-class accuracies of 70% (14/20) and 74% (14/19), respectively. In contrast, the ddH_2_O group had a per-class accuracy of 50% (10/20, Fig. 1d, e). In predicting the immunizations, including all treatments, the βgal group showed a 63% per-class accuracy (22/35 samples), whilst 77% per-class accuracy for the TSHR (33/43, Fig. 1f, g). Similar outcomes were retained when repeating the model without the vancomycin-treated group (Figure S1D–G).

### Combined effect of treatments, immunizations and time on the fecal microbiota composition of GO mouse model

**Fig. 2:**
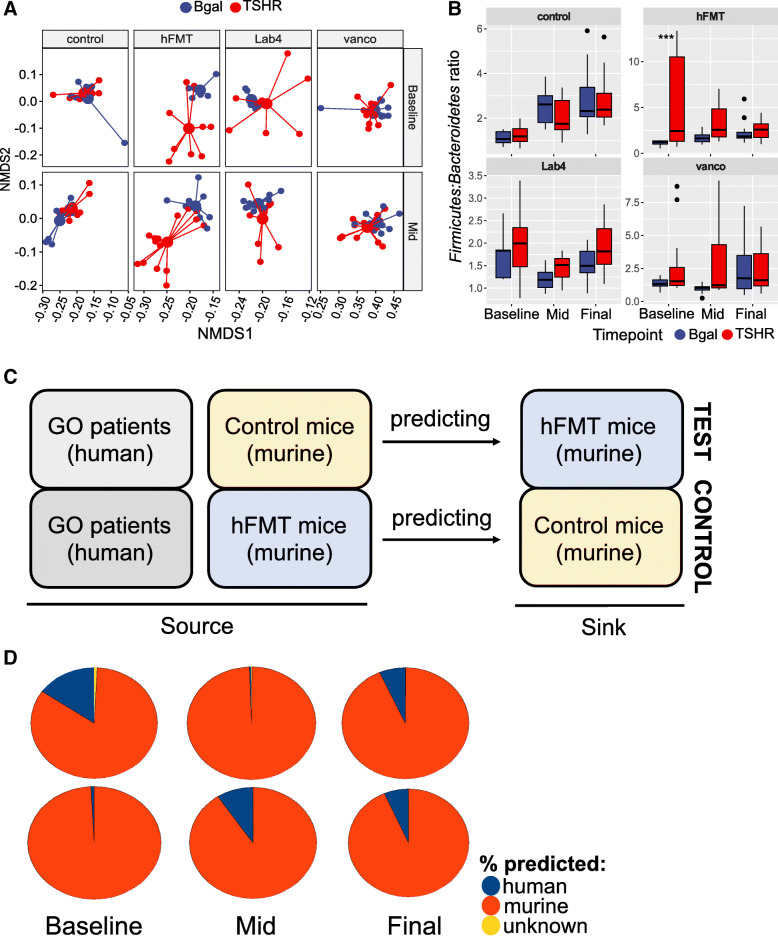
Effect of treatments, immunizations and time on the GO model gut microbiota. **a** Differences between immunizations at each timepoint and per treatment. PERMANOVA with 999 permutations, ***P = 0.008 and **P = 0.016 (*n* mice/treatment/immunization at baseline: control TSHR = 10, βgal = 6; hFMT-TSHR = 9, βgal = 6; Lab4 TSHR = 9, βgal = 5; vancomycin TSHR = 12, βgal = 8) (*n* mice/treatment/immunization at mid-timepoint: control TSHR = 11, βgal = 9; hFMT-TSHR = 5, βgal = 9; Lab4 TSHR = 11, βgal = 11; vancomycin TSHR = 14, βgal = 4). **b** Firmicutes to Bacteroidetes ratio comparing immunizations in each timepoint and within each treatment group. Welch’s *t* test with BH correction: ***P = 0.0006. **c** Rationale of the SourceTracker analysis (see STAR methods). Human GO donors (*n* = 6), murine controls (*n* control/timepoint: baseline = 16, mid = 20, final =20) and hFMT-receiving mice (*n* hFMT/timepoint: baseline = 15, mid = 24, final = 19). **d** Pie charts: engraftment expressed as average % probability using the control and the test analysis in each timepoint. Unknown: observations not assigned to a specific source at the significant threshold (*a* = 0.001)

We assessed the impact of various treatments over time by analysing the fecal microbiota after gavage but prior to any immunization (baseline) and following 3 gavages but before the third immunization (midpoint) (Fig. 1a). At baseline, vancomycin treatment already produced drastic effects, comparable to those reported at the end of the experiment (Figure S5A). Compared to ddH_2_O-controls, Lab4 increased both Chao1 (Figure S5A) and observed OTUs indices, whilst hFMT reduced bacterial diversity (Shannon) and evenness, but did not reach significance. Lab4- and hFMT-treated mice displayed differences in fecal microbiota composition compared to ddH_2_O- and vancomycin-treated mice (i.e. reduced counts of *Bacteroides* spp*.*), but the two groups differed very little at baseline (Figure S5B). A significant difference in the Firmicutes to Bacteroidetes ratio was observed between TSHR and βgal immunizations in the hFMT-receiving mice (*P* = 0.0006, Fig. 2b). Similarity between hFMT-receiving mice and the GO human donors (Fig. 2c) was calculated with the SourceTracker algorithm [39] using the family-level microbiota composition. At baseline, 4 out of 15 hFMT-receiving mice fecal samples (test) showed more than 40% similarity (min 46%, max 72%) with the GO donors’ source, whilst none out of 16 ddH_2_O-receiving mice (control) shared any similarity with the GO donors samples (Fisher’s exact test with Yates’ continuity correction, *P* < 0.001; Fig. 2d).

At midpoint, diversity and evenness (but not richness) of the vancomycin-treated gut microbiota further reduced compared to baseline. Conversely, richness indices significantly increased between baseline and midpoint in TSHR-hFMT mice (*P* = 0.023 observed OTUs and *P* = 0.019 Chao1, respectively), as in the ddH_2_O group (*P* = 0.014 observed OTUs and *P* = 0.0038 Chao1; Figure S5C). Bray-Curtis-based non-metric dimensional scaling (NMDS) separation between hFMT and Lab4 mice (considering the two immunizations together) was significant (Fig. 2a).

No significant differences in beta-diversity were observed between TSHR and βgal immunizations at baseline, except in the hFMT-treated mice (*P* = 0.008). which lasted at the midpoint (*P* = 0.016, Fig. 2a). Centroids (sampling distribution of the mean) of each immunization were close to each other, replicating our previous findings at the equivalent T2 timepoint (Masetti et al., 2018). *Bacteroides* spp. significantly increased with time in Lab4 mice, in both immunizations (data not shown). At midpoint, none of the hFMT-receiving mice (0/24) showed any similarity with the human donors (*P* < 0.001) whilst half of the ddH_2_O mice (10/20) showed > 10% similarity of the gut microbiota with GO donors (Fig. 2d). At the end of the experiment, the same similarity to human donors was observed in both murine controls (26%) and hFMT-receiving colon samples (26%).

### Influence of gut microbiota modifications on TSHR directed autoimmunity

**Fig. 3:**
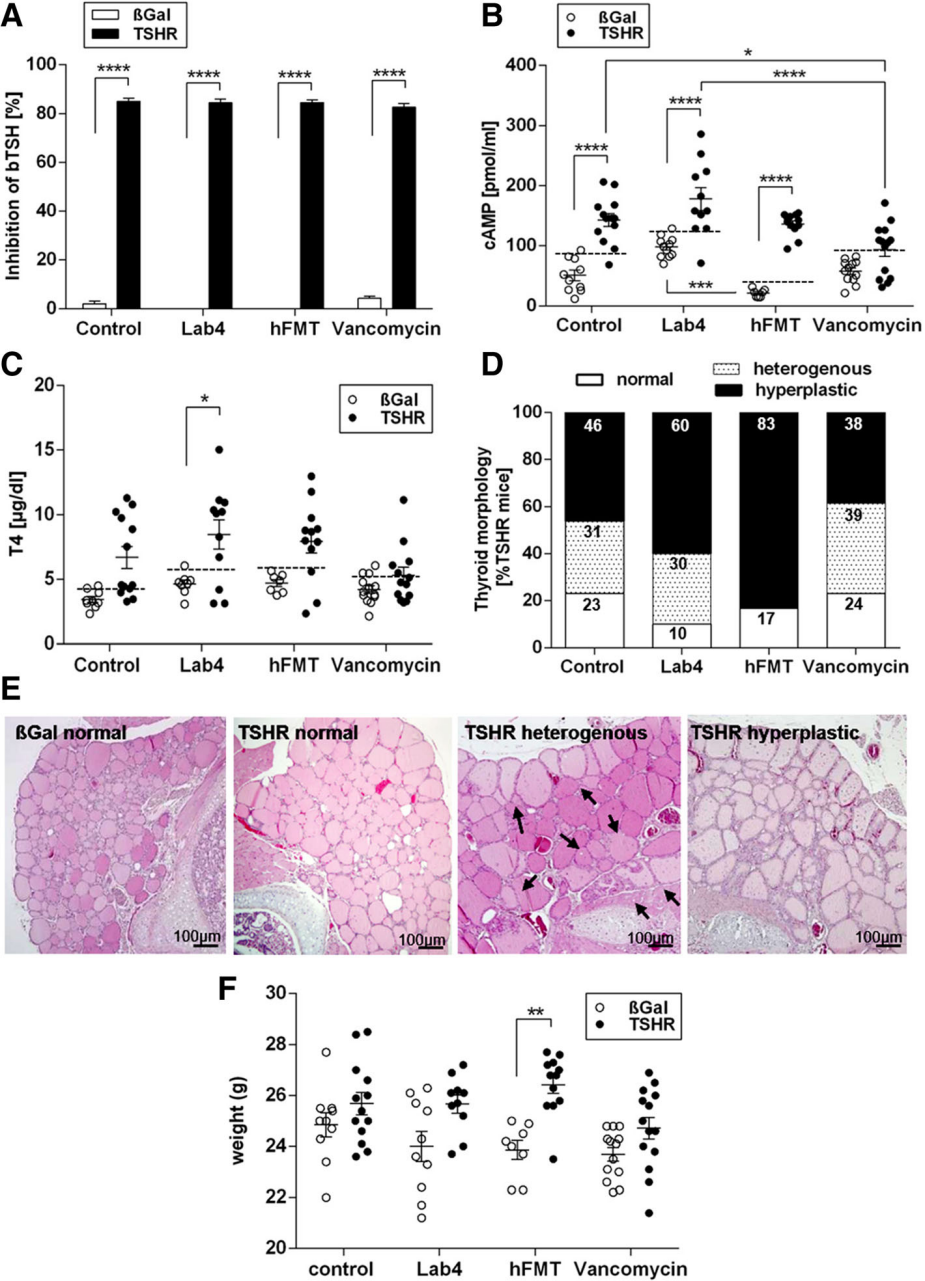
Mouse TSHR antibody evaluation and impact of gut microbiome modification on thyroid function and morphology. **a** Total TSHR antibodies (TRAbs) were measured by TSH binding inhibitory immunoglobulin activity to the human TSHR given as % inhibition of bTSH. **b** Thyroid-stimulating autoantibodies (TSAbs). Stimulating activity is given in cAMP (pmol/mL). Data are presented as mean ± standard error of the mean. Multiplicity adjusted *P* values are marked as follows ****P < 0.0001, ***P < 0.001, *P < 0.05 (two-way ANOVA). **c** Total T4 values (μg/dl) in βgal- and TSHR-immunized mice from each treatment group *P < 0.05. **d** H&E of thyroid slices for thyroid morphology evaluation (in normal, heterogeneous (hetero) or hyperthyroid (hyper)). **e** Representative thyroid images at × 20 magnification. Heterogeneous thyroids contained normal and hyperthyroid regions (indicated by arrows). **f** Weights of animals at the endpoint of the experiment. Data are presented as mean ± standard error of the mean. Multiplicity adjusted *P* value is marked as **P < 0.01 (two-way ANOVA)

Antibodies to the human TSHR were measured by Thyrotropin binding inhibiting immunoglobulins (TBII; TRAb) and were significantly detected in all TSHR-immunized mice, but not in βgal-ddH_2_O mice (*P* ≤ 0.0001), indicating successful immunization (Fig. 3a). Thyroid-stimulating antibodies (TSAb) were measured using a mouse TSHR overexpressing CHO cell-based bioassay. ddH_2_O-, Lab4- or hFMT-treated TSHR-immunized mice showed a statistically significant induction of TSAb compared to respective βgal mice (*P* ≤ 0.0001) but not in the vancomycin-treated group (Fig. 3b). We also quantified percentages of mice developing significant TSAB based on the upper 99% CI of the respective βgal mouse group as the positive threshold (Table S2). Eighty-four percent of TSHR-ddH_2_O mice developed significant TSAB; treatment with Lab4 or hFMT increased positivity to 90% and 100%, respectively, but vancomycin treatment reduced TSAB to 64.3% of mice (Table S2).

Disease features were correlated with the bacterial biomarkers identified by the two random forest models by Pearson’s correlation coefficient. In TSHR-ddH_2_O, six genera including *Parabacteroides* counts correlated positively with TRAbs. Positive correlations were also observed between TRAB and counts of unidentified/uncultured Firmicutes in TSHR-vancomycin mice and with *Parabacteroides* in TSHR-hFMT. Negative correlations were seen in TSHR-hFMT mice between TRAB and *Lactobacillus* and in TSHR-Lab4 between TSAB and uncultured Firmicutes, *Lachnoclostridium* and uncultured Bacteroidetes (Figure S6) and a strong negative correlation with the genus *Lactobacillus*.

### Effect of microbiome modification on thyroid function

Total T4 values tended to increase in TSHR-immunized groups, with Lab4-treated showing significantly higher T4 compared to the corresponding βgal (*P* ≤ 0.05) (Fig. 3c). Based on the 99% CI of the relevant βgal-immunized mice, 66.7% of TSHR-ddH_2_O mice had elevated T4 levels which increased to 73% and 75% in Lab4- or hFMT-treated respectively but reduced to 36% in vancomycin mice (Table S2). Histological examination of the thyroid revealed variable hyperplastic morphology (thickened epithelium, reduced colloid) indicating gland hyperactivity in TSHR-immunized mice but not in the βgal groups (Fig. 3d, e). Some thyroids had heterogeneous morphology with normal regions and areas of hyperplastic follicles (Fig. 3e, arrows). Hyperplastic/heterogeneous morphology was found in 46% and 31% respectively, of TSHR-ddH_2_O mice (Fig. 3d). Lab4 and hFMT increased hyperplastic thyroid to 60% and 83%, respectively (Fig. 3d). The hFMT morphology paralleled their increased TSAb and elevated T4 levels when compared with other groups (Table S2). In contrast, vancomycin reduced hyperplastic thyroids to 38% with 39% having heterogeneous morphology, suggesting milder hyperactivity of the gland compared to other mice (Fig. 3e, Table S2). In addition, weights of hFMT-treated TSHR-immunized mice were significantly increased compared to βgal-immunized mice (Fig. 3f).

TSHR-ddH_2_O mice showed positive correlations between T4 and the same three Firmicutes genera correlating with TSAB. Positive and negative correlations were observed between T4 and *Lachnoclostridium* and the genus *Ruminiclostridium* in vancomycin- and Lab4-treated, respectively (Figure S6).

### Microbiome modification modulates Graves’ orbitopathy

**Fig. 4:**
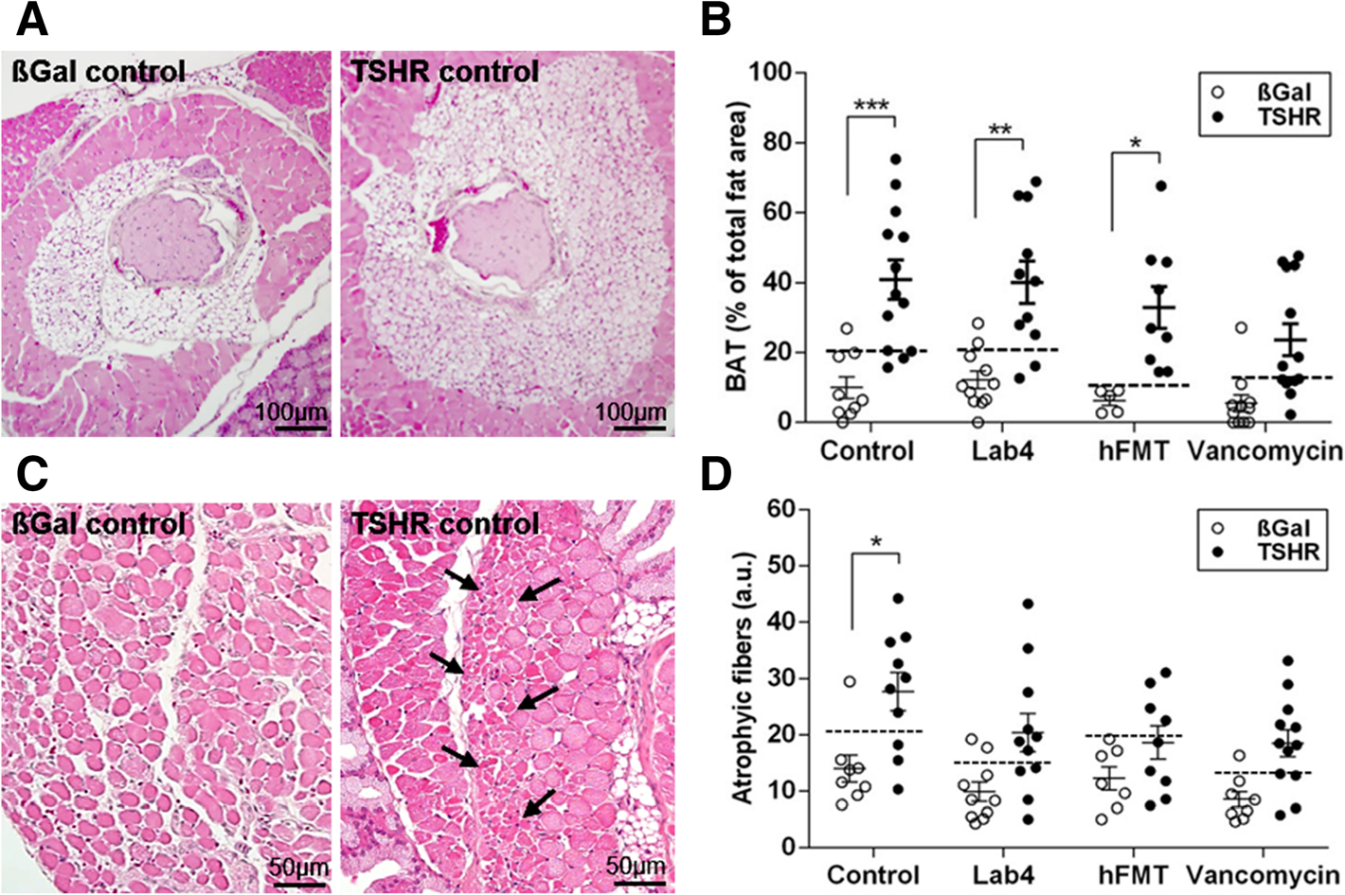
Gut microbiome modification modulates orbital tissue abnormalities in GO mouse model. The orbits of mice were fixed, paraffin embedded, and consecutive slices of the middle orbital area were stained with H&E and evaluated for **a** brown adipose tissue (BAT); representative images are shown, magnification × 10. **b** BAT as the percentage of the total fat area. **c** Atrophic fibers in extra orbital muscles; representative images are shown, magnification × 20, arrows indicate areas with atrophic fibers. **d** atrophic fibers/total muscle fiber area (a.u.). Data are presented as mean ± standard error of the mean. Multiplicity adjusted *P* values are marked as ***P < 0.001, **P < 0.01, *P < 0.05 (two-way ANOVA)

Individual TSH-immunized mice developed eye signs, e.g. proptosis and inflammation, indicating orbitopathy to different degrees which were difficult to quantify objectively (Suppl. Figure S7A). To determine the impact of gut microbiome modification on orbitopathy, histological sections of mice orbits were analysed. We have previously shown that the mouse orbit contains TSHR-positive brown adipose tissue (BAT) which is enlarged in the GO model [40–42]. Orbital BAT is characterized by small fat lobules and can be distinguished from orbital white adipose tissue (Fig. 4a, Suppl. Figure S7B). In agreement with our earlier studies, BAT area increased in the orbital tissue of TSHR-ddH_2_O mice (69% positive mice based on 99% CI) compared to βgal-ddH_2_O mice (*P* ≤ 0.001; Fig. 4b). Enlargement of BAT was enhanced in TSHR-immunized mice treated with Lab4 (*P* ≤ 0.01; 81.8% positive mice) or hFMT (100% positive mice). In contrast, vancomycin-treated TSHR-immunized mice had a much lower portion of BAT positive mice (53.8%) compared to the other TSHR-immunized mouse groups (Table S2). Previously, we described muscle atrophy (smaller muscle fibers, Fig. 4c arrows) as another feature of orbitopathy in TSHR-immunized mice [27]. Significant muscle fiber atrophy was detected in TSHR-ddH_2_O mice compared to βgal-ddH_2_O group (*P* ≤ 0.05) and not in the other three groups (Fig. 4d). Based on 99% CI, 70% of TSHR-ddH_2_O mice developed significant muscle atrophy whilst 63.6% of Lab4-, 44.4% of hFMT- or 66.7% of vancomycin-treated TSHR-immunized mice had some muscle atrophy (Table S2). However, lymphocytic infiltrations were not detected in the orbital tissues (Suppl. Figure S7C, D).

In the vancomycin-treated TSHR-immunized group, a strong negative correlation was observed between the total adipose value, BAT and the *Akkermansia* spp. and between *Bacteroides* spp. and the total adipose tissue whilst a positive correlation was observed between *Lachnoclostridium* counts and BAT. In Lab4-TSHR, atrophy correlated negatively with *Lachnoclostridium* spp. and uncultured Firmicutes as well as uncultured Bacteroidetes, whilst *Akkermansia* spp. correlated negatively with the total adipose tissue (Figure S6).

### Modification of the gut microbiome affects disease features to varying degree

**Fig. 5:**
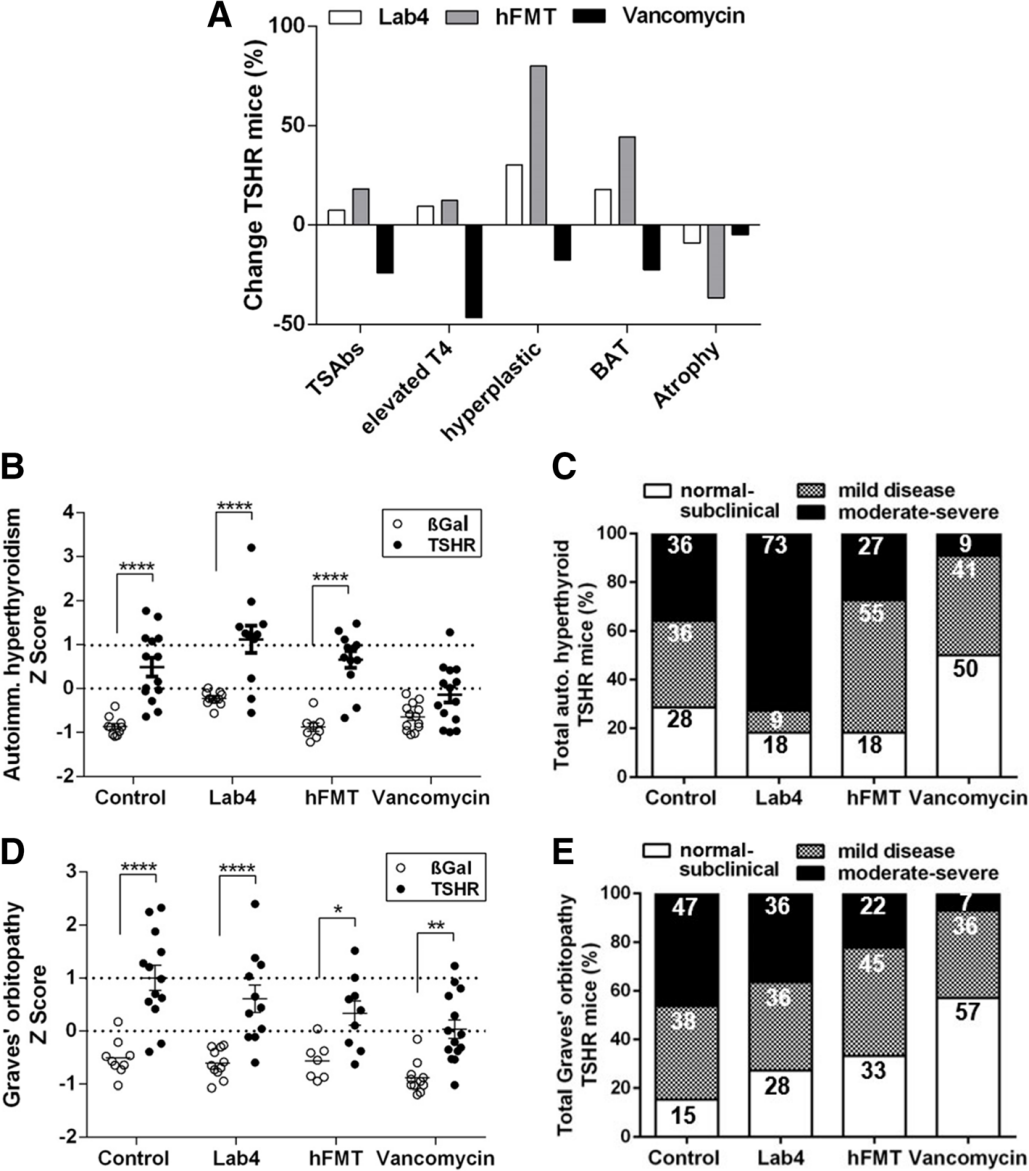
Impact on autoimmune hyperthyroidism and orbitopathy and total disease outcomes of gut microbiota modifications. **a** Percentages of positive untreated TSHR-immunized mice set to 100% and the changes of positivity in the treated groups Lab4, hFMT or vancomycin were calculated. Changes in positive mice are given in % relative to untreated TSHR-immunized mice. Parameters were normalized using the Z-Score (see the “Materials and methods” section). **b** Z-Score of autoimmune hyperthyroidism includes values of TSAbs and T4. **c** Z-Score of orbitopathy evaluated by BAT and atrophic fibers. Data are presented as mean ± standard error of the mean. Statistical analysis was performed by two-way-ANOVA; multiplicity adjusted *P* values are given as follows: *P ≤ 0.05; **P ≤ 0.01; ***P ≤ 0.001; ****P ≤ 0.0001. **d**, **e** Disease classification was based on Z-score values. The number of mice is given in %, see STAR methods for classification parameters. Additionally, the number of mice in each category is shown in Table S2

To compare the effects of the various microbiota modification treatments on characteristic disease features, the percentages of mice positive for TSAbs, elevated T4, hyperplastic thyroid morphology, BAT and muscle atrophy of each TSHR-immunized mouse group were evaluated based on the upper 99% CI of the respective βgal mouse group (Table S3). Percentages of positive TSHR-ddH_2_O mice were set to 100% and the relative changes of positivity in the treated groups were calculated (Fig. 5a). Lab4 and hFMT, both increased the percentage of mice positive for TSAb, elevated total serum T4 and orbital BAT enlargement, but reduced mice with orbital muscle atrophy. hFMT also strongly increased percentages of mice with hyperplastic thyroid morphology. Vancomycin reduced the percentage of mice positive for TSAb, elevated T4, hyperplastic morphology and orbital BAT whilst orbital atrophy was only mildly affected (Table S2 and Fig. 5a).

### Modification of gut microbiome determines the total outcome of disease

**Fig. 6:**
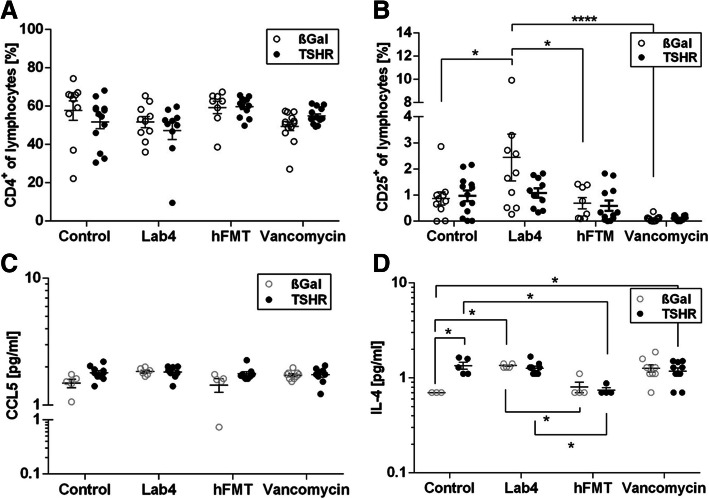
Effects of gut microbiome modification on lymph node T cell subsets, cytokine and chemokine levels. **a** CD4^+^ and **b** CD25^+^ T cell population from draining lymph nodes of TSHR- and βgal-immunized mice in each treatment group. Data are presented as mean ± standard error of the mean. Multiplicity adjusted *P* values are marked as ****P < 0.0001, *P < 0.05 (two-way ANOVA). **c** CCL5 and **d** IL-4 circulating chemokines/cytokines from sera. Cytokine values are log10 transformed. Multiplicity adjusted *P* values are marked as *P < 0.05 (two-way ANOVA).

The differently treated TSHR-immunized mouse groups developed autoimmune hyperthyroidism or orbitopathy to varying degrees. To compare total disease outcome between the mouse groups, we normalized and combined the different parameters for each mouse by the Z-score method as described earlier [27, 35, 42]. As shown in Fig. 6, the TSHR-ddH_2_O mouse group (Z-score > 0) can be clearly separated from βgal mouse group (Z-score 0) concerning autoimmune hyperthyroidism (*P* ≤ 0.0001) and orbitopathy (*P* ≤ 0.0001) (Fig. 5b, c). Thus, TSHR-ddH_2_O mice manifested significant overt disease (termed clinical disease). Z-score values between 0 and 1 were defined as mild disease and Z-scores > 1 were regarded as moderate-to-severe disease as described previously [42]. TSHR-immunized mice with Z-score < 0 were classified as normal-to-subclinical disease, i.e. have only developed TSHR antibodies (Fig. 5b). Numbers of mice showing normal-to-subclinical, clinical, mild or moderate-to-severe disease are given in Table S3. Overall, hFMT and Lab4 increased the incidence and severity of autoimmune hyperthyroidism whereas vancomycin treatment led to mild or no disease compared with TSHR-ddH_2_O (Fig. 5d). Regarding orbitopathy, 85% of TSHR-ddH_2_O, 72% of Lab4 and 67% of hFMT mice developed either mild or moderate-to-severe disease. In contrast, only 43% of vancomycin-treated TSHR-immunized developed mostly mild GO (Fig. 5e).

### Microbiome modification changes profile of lymphocytes

Flow cytometry analysis of orbital draining lymph node revealed that CD4^+^ lymphocyte numbers remained unaffected (Fig. 6a), whilst CD4^+^CD25^+^ (regulatory T cells; Tregs) numbers were significantly lowered in vancomycin-treated TSHR- and βgal-immunized mice (Fig. 6b) suggesting lower autoimmunity incidence and disease severity that we observed in this group is not necessarily regulated by local mechanisms. Lab4-treated βgal mice showed significantly higher Tregs compared with the ddH_2_O and hFMT-βgal groups. However, such an elevation in Tregs was not observed in Lab4-treated mice when they underwent induction of GD/GO by receiving TSHR immunization. TSHR-ddH_2_O mice showed positive correlations between CD4^+^CD25^+^ levels and *Bacteroides* and *Alistipes* genera and between Lachnospiraceae family. CD4^+^ levels negatively correlated with six genera, including *Bacteroides* and *Parabacteroides* spp. In the vancomycin-TSHR immunization group, *Akkermansia* spp. positively correlated with CD4^+^CD25^+^ levels, whilst an uncultured Bacteroidetes was positively correlated with CD4^+^ T cells. In Lab4-TSHR, genus *Akkermansia* negatively correlated, whilst *Alistipes* spp. positively correlated with CD4^+^CD25^+^ levels. Uncultured Bacteroidetes correlated positively with CD4^+^CD25 and negatively with CD4^+^. Uncultured Actinobacteria displayed a strong negative correlation to CD4^+^, as for *Bacteroides* and *Parabacteroides* spp. In hFMT-TSHR, genera *Bacteroides* and *Alistipes* negatively correlated to CD4^+^ (Figure S6).

### Microbiome modification affects circulating cytokine and chemokine levels

Chemokines and cytokines were measured in sera at sacrifice using a multiplex system. Several were below the limit of detection (IL-17/17A, IL-2 and IFN-γ), and no significant differences were seen in IL-10, CCL20, IL-6 or TNF-alpha. The chemokine CCL5/RANTES showed a trend to increase in TSHR-immunized compared with βgal only in ddH_2_O treated mice (*P* = 0.37) whilst IL-4 was significantly higher in TSHR-ddH_2_O-compared with βgal (*P* = 0.0170) (Fig. 6c). Vancomycin treatment increased IL-4 levels significantly in βgal mice compared to ddH_2_O treatment (*P* = 0.0286) and in βgal-immunized mice compared to hFMT-TSHR mice (*P* = 0.0228) which correlated with Verrucomicrobia and Firmicutes. In TSHR-Lab4 IL-4 increased compared to hFMT (*P* = 0.0208) (Fig. 6d).

Significant positive correlations between CCL5 and two unidentified and uncultured Firmicutes were observed in TSHR-ddH_2_O mice. A positive correlation between IL-4 and *Akkermansia* spp., a negative correlation with *Lachnoclostridium* spp. Lab4-TSHR and a positive correlation between CCL5 and *Parabacteroides* spp. were reported in TSHR-vancomycin mice (Figure S6).

## Discussion

The current study extends our previous work in which we demonstrated significant differences in microbiota composition from inbred BALB/c mice, undergoing the same immunization protocol, in specific pathogen-free (SPF) units in different locations. The results were obtained using a robust GD/GO mouse model and supported our hypothesis that the bacterial environment may contribute to determining TSHR-induced responses and hence influence the outcome and reproducibility of induced GD and GO.

For the first time, we investigated whether the gut microbiota is necessary for the induction of GD/GO, by modifying its composition from early-life and prior to the hTSHR immunization regimen. Modulation of the gut microbiome has been reported to improve or exacerbate other autoimmune diseases. We obtained striking results: antibiotic treatment had a profound impact on the gut microbiome which was associated with significantly reduced GD-like disease (pathogenic autoantibodies, elevated thyroxine, hyperthyroid thyroid morphology) and GO-like histopathology and cellular changes (orbital adipogenesis and muscle atrophy). In contrast, mice receiving hFMT from patients with severe GO, initially shared similarity of their gut microbiota composition with their donors, accompanied by increased severity of GD-like disease. The fact that muscle atrophy decreased but orbital BAT volume was increased in hFMT recipients suggests different pathogenetic mechanisms for these features of GO. Mice receiving probiotic treatment had significantly higher numbers of CD25^+^ Tregs in orbital draining lymph nodes.

Consistent with previous findings [43], the effects of long-term vancomycin treatment were dramatic and included depletion of the richness and diversity indices accompanied by reduction of Gram-positive bacteria (mainly Firmicutes phylum) and an increase in Proteobacteria species, including *Salmonella* spp*., Pseudomonas* spp. and *E. coli*. Vancomycin also fostered growth of resistant and/or compensating species, e.g. the highest counts of the genus *Bacteroides*, which correlated negatively with total orbital fat and genus *Akkermansia* which showed a significant negative correlation with brown and total fat, CD4^+^ and CD8^+^ in the orbit (data not shown) and a positive correlation with the CD25^+^ (Tregs) in TSHR-immune mice. *Akkermansia muciniphila* constitutes a single-species of the genus *Akkermansia* [44], which is involved in mucin degradation [45]. The postnatal administration of vancomycin in non-obese diabetic (NOD) mice reduced the incidence of T1D along with the increased proportion of *A. muciniphila* [46], although other studies showed exacerbation of T1D in murine models and human subjects in the presence of antibiotics [47, 48].

At the start of immunization, some hFMT mice had a GO-like environment in their gut. In particular, mice showing the highest engraftment were housed in the same cage, suggesting a possible cage effect. However, we repeated the analysis removing the “highly-colonized” mice and similar results were confirmed (Figure S4). At the midpoint (after 6 weeks of washout), no similarity between hFMT-receiving mice and GO donors was observed, whilst at the end of the experiment, the large intestines of both ddH_2_O and hFMT mice shared the same similarity with human samples. The algorithm used OTUs at the family level, which may lack host specificity, since no similarity was observed between mice and humans at lower taxonomies. The definition of FMT success is primarily based on a positive clinical response in the recipient. However, from a microbiological perspective, FMT success can also be defined by a shift in the gut microbiome profile of an individual towards that of the donor. We argue that a successful engraftment may be a two-step process; first requiring the transplanted microbiome to engraft within the new host and augment the local commensal community, after which clinical response may be observed.

The washout period may have reduced the transferred bacteria, and the lack of prior treatment, such as bowel cleansing or antibiotic treatment of dams, despite pups being gavaged from birth, may have allowed some transmission of maternal gut microbiota, which can induce colonization resistance [49]. Of note, freeze-dried FMT proved to be a safe and efficient treatment of diarrheal episodes in recurrent *Clostridioides difficile* infections, often accompanied by an increased/restored microbiota diversity and a successful engraftment [50–52]. In mice, after bowel cleansing, the engraftment of a single FMT gavage lasted up to 4 weeks, whilst repeated gavages (i.e. twice a week for 4 weeks) negatively impacted the stability of the gut microbiota [53]. Furthermore we used fecal material from people with severe GO, our parallel studies in patients with GD and/or GO reveal that most significant differences in gut microbiota appeared in mild GO, compared with GD or healthy controls. Bacterial abundance and diversity of patients with severe GO more closely resembled that of healthy controls, possibly due to the extensive treatments, including corticosteroids, experienced by this group.

Probiotic supplements are associated with health benefits in humans, including immune-modulation of the recipient [54], and this was demonstrated in our Lab4-treated mice. Specifically, CD4^+^CD25^+^ Tregs were induced in the βgal, but not in the TSHR-immune group, although the latter showed interesting correlations, e.g. Bacteroidetes-uncultured genera, *Bacteroides* and *Alistipes* negatively correlated with CD4^+^ and simultaneously positively with CD4^+^CD25^+^ Tregs. This suggests that although probiotic treatment increases local Tregs, this is unable to prevent breakdown in tolerance following TSHR immunization.

The significantly lower CD4^+^CD25^+^ Tregs numbers in vancomycin-treated mice in our study are in line with previous findings. Vancomycin selectively targets gram-positive bacteria which produce the short-chain fatty acid butyrate [55]. Butyrate promotes the differentiation of Tregs from naive CD4^+^ T cells and is known for its anti-inflammatory effects [56].

Beneficial effects conferred by probiotics are not always related to the effective colonization of the host mucosa by probiotic species and also occur transiently [57]. Therefore, it was not surprising that no increase of *Lactobacillus* sp. was observed in Lab4-treated LGI microbiota. However, *Bifidobacterium* sp. was not detected at all from the gut microbiota of our BALB/c mice (even before the removal of low abundant OTUs), as previously reported [25, 27, 28], possibly due to environmental factors (e.g. animal housing) or because of a poor colonizer setting.

Lab4-treated TSHR-immune mice showed significantly higher T4 levels and orbital brown fat compared to βgal. Varian and collaborators treated aging outbred mice daily with *Lactobacillus reuteri* and reported increased T4 levels, accompanied by weight loss and increased activity compared to untreated. Authors also observed an enlarged thyroid and induced activity dependent upon CD4^+^CD25^+^ Tregs [58]. Administration of *L. acidophilus* for 32 days increased TSH and T3 levels but not T4 in weaning rats [59]. Whilst providing a “healthful aging” in 1-year-old mice [58], it is possible that probiotics worsen hyperthyroidism following TSHR immunization. On the contrary, supplementation of *Bifidobacterium lactis* and *Lactobacillus rhamnosus* mitigated disease outcome in experimental autoimmune thyroiditis (similar to Hashimoto’s thyroiditis) [11]. In Hashimoto’s thyroiditis patients, administration of probiotics stabilized hormonal fluctuations during therapy, although no significant protective effects were observed in hyperthyroid humans compared to healthy controls [60]. In other autoimmune conditions, prevention of T1D onset was observed in NOD mice receiving multiple strains of *Lactobacillus* and *Bifidobacterium* spp. and of *Streptococcus salivarius* subsp. *thermophilus* which was associated with increased production of anti-inflammatory IL-10 [61]. Similarly, administration of probiotics reversed the EAE model phenotype with an upregulation of Tregs via IL-10 production [62].

The study also shows some limitations. As mentioned above, hFMT was not accompanied by any pre-treatment. Usage of freeze-dried material would have probably been more effective if we obtained samples from mild GO rather than sight-threatening disease, particularly since patients had gone through massive interventions before donating fecal samples. If and how each of those treatment regimens has influenced patients’ gut microbiota is not completely clear. Moreover, despite administering a probiotic containing two bifidobacteria species, we were unable to detect them in any of the mice studied, for the reasons already mentioned.

The fact that TSHR-immunized mice gained weight was a surprise and contrasts with GD patients who lose weight during the active stage of disease, although most gain weight when TRAb-positive and euthyroid [63]. However, other than in patients, female mice were shown to gain weight in hyperthyroidism induced by T4 treatment, most likely due to higher food intake [64]. Likewise, in a recent study, we showed that hyperthyroid TSHR immune mice gain weight [42]. Consistently, in the current study, we found that weight gain correlated positively with T4 values or with TSAb in the TSHR immune mice but not in the βgal-ddH_2_O mice (Figure S9). Of note, hFMT mice showed the most significant weight gain suggesting a strong hyperthyroid hormone state during the experimental course. Indeed, hFMT mice showed the strongest elevated hyperactive thyroid morphology confirming this assumption. Induction of hyperthyroidism was highly variable in the mice, whether assessed by T4 measurement or thyroid morphology. The heterogeneity of autoimmune thyroid dysfunction in the model reflects the situation in patients [26, 35]. Most GO patients had developed hyperthyroidism when GO is diagnosed but some GO patients developed GO although euthyroid or even hypothyroid [65]. Whether and to what extent the thyroid hormone level directly impacts/modulates the outcome of GO in the model is unclear and needs to be addressed in further studies.

Our findings are relevant to human disease, as illustrated by the similar perturbations of the gut microbiota (increased Firmicutes and decreased Bacteroidetes) we have found in TSHR-immunized mice and GD/GO patients (ms in preparation). Furthermore, long-term vancomycin treatment resulted in a reduced and resilient microbiota, which included high counts of *Bacteroides* spp. (the opposite of that found in murine and human GD/GO) and *Akkermansia* spp. Such an antibiotic-modified-gut microbiota reduced incidence and severity of disease induced by subsequent TSHR immunization. In contrast, hFMT exacerbated GD-like pathology, i.e. the disease phenotype had been partially transferred, and was associated with the lowest *Bacteroidetes* counts of all four TSHR-immunized groups. Whether increased Firmicutes or decreased Bacteroidetes has more effect on pathogenesis is not clear; some disease states have been associated with gain of function perturbation of the gut microbiota, e.g. colorectal cancer and Parkinson’s disease whereas others have been associated with loss of function imbalance, e.g. inflammatory bowel disease. Of note, in most chronic conditions, decreased microbiota diversity is a common theme [66].

The lack of induced disease in the vancomycin-treated TSHR-immune mice confirms that the gut microbiota is necessary for GD/GO to be successfully induced, potentially training the immune system at the early stage of life. Ivanov and collaborators reported a decreased Th17-produced proinflammatory cytokines milieu in the small intestines of the EAE newborn pups treated with vancomycin, which may have contributed in the protection from the disease development [67]. A very recent paper by Su et al. reported significantly reduced SCFA-producing bacteria in GD patients. Furthermore, propionic acid from *Bacteroides* spp increased Tregs whilst reducing Th17 cells [68]. This ties in with studies from Fang and colleagues who identified a significantly higher proportion of IL-17A-producing T cells in GO patients and which correlated with orbital fibrotic change [69]. In our experiments, circulating IL-17/17A, IL-2 and IFN-γ levels were below the limit of detection, although cytokine measurements were possible only at the end of the experiment. In a few TSHR-immunized mice, elevated levels of IL-10, IL-6, CCL-20 and TNF-alpha were observed (data not shown). However, we did observe a trend towards increased CCL5/RANTES levels in TSHR-immunized mice, a chemokine which is released by orbital fibroblasts leading to T cell migration and inflammatory responses [70]. IL-4, a cytokine produced by Th2 cells which is increased in serum of patients with GD [71] and which has mitogen effects on fibroblasts [72], was found to be increased in TSHR-ddH_2_O mice and in βgal mice after treatment with Lab4 or vancomycin. We did not confirm the reported reduction of IL-4 by treatment with probiotics [73] although our findings agree with Sun and colleagues who showed upregulation of IL-4 gene expression after vancomycin treatment. Alternatively, since the TSHR-induced model does not employ conventional adjuvants, given its close interplay with the immune system, the gut microbiota can itself act as a natural adjuvant, promoting (or not) the second immune stimulus needed for the activation of the autoimmune response, as reported by Oh and co-workers [74].

We speculate that the Lab4 administration promoted an anti-inflammatory response, increasing the Tregs in the βgal-ddH_2_O mice, which was neutralized by the TSHR immunization, despite gut microbiota-correlating features. Also, the lack of induced disease in vancomycin-treated mice was accompanied by the lack of Tregs in both βgal and TSHR mice. Future studies in which immune cells are transferred from probiotic-treated mice to naive recipients, prior to TSHR immunization, will facilitate identification of the T cell subsets implicated in GD/GO.

## Conclusions

In conclusion, our results strongly support a pivotal role for the gut microbiota in TSHR-induced disease (Suppl. Figure S10). Whilst changes to the gut microbiota have a profound effect on quantifiable GD endocrine and immune factors, the impact on GO cellular changes is more nuanced. Future studies will address the translational potential of our work, including clinical trials of appropriate antibiotics, which are warranted to improve outcomes for patients with GD/GO. Also, a placebo-controlled randomized trial with probiotics has been conducted in patients to investigate the potential protective effect on hormonal fluctuations on GD/GO during anti-thyroid treatments.

## Supplementary Information

**Additional file 1: Supplementary methods. Table S1**. Characteristics of patients with sight-threatening GO recruited at the University Hospital Duisburg-Essen providing samples for hFMT production.

**Additional file 2: Figure S1**. Supplementary results for the endpoint gut microbiota analysis. (A and B) Microbiota composition according to different anatomical samples in TSHR and βgal-immunized mice (samples/source: colon = 48, entire = 30, small = 51 from final timepoint and stool = 105 from baseline and mid-timepoint). (A) Alpha-diversity indices of the source of the microbiota sampled, Wilcoxon-Mann BH corrected test: ***P < 0.05. (B) NMDS of Bray-Curtis distances according to immunizations and sources at the endpoint. PERMANOVA between entire-colon samples *P* > 0.05. (C and D) Endpoint composition of the LGI microbiota amongst treatments in βgal-immunized mice. (n βgal mice/treatment at endpoint: control = 8, hFMT = 8, Lab4 = 10, vancomycin = 19). (C) Alpha diversity amongst treatments, Wilcoxon-Mann BH corrected test:***P < 0.001. (D to G) RandomForest of a model excluding vancomycin samples (n mice/treatment endpoint: control = 20, hFMT = 19, Lab4 = 20). (D) Confusion matrix for treatments w/o vancomycin samples. Diagonal boxes represent the number of samples correctly predicted. (E) Top-10 variables of treatment classification according to the Mean Decrease Gini, including the microbiota source as an effect related to figure E. (F) Confusion matrix for immunizations in a model w/o vancomycin samples. (n mice/immunization endpoint: TSHR = 33 and βgal = 26). (G) Top-10 variables of immunizations classification according to the Mean Decrease Gini, including the microbiota source and treatments as an effect related to figure G. Wilcoxon-Mann test with BH correction: ***P < 0.005; **P < 0.01; *P < 0.05.

**Additional file 3: Figure S2**. Heatmap of the differentially abundant genera amongst treatments in TSHR-immunized mice. Median abundances were scaled according to row Z-score. Only genera with *P* < 0.5 are represented.

**Additional file 4: Figure S3**. Heatmap of the differentially abundant genera amongst treatments in βgal mice. Median abundances were scaled according to row Z-score. Only genera with *P* < 0.5 are represented.

**Additional file 5: Figure S4**. Composition of the fecal microbiota in GO patients donating the samples for the hFMT (*n* = 6) and healthy controls from the same region (*n* = 12). (A) Chao1 alpha-diversity index. Wilcoxon-Mann test, *P* = 0.80. (B) Firmicutes to Bacteroidetes ratio. Wilcoxon-Mann test, *P* = 0.60. (C) Heatmap of the top-50 most abundant genera in GO patients and healthy controls. Each column represents a sample. Relative abundances were scaled according to the row Z-score. (D) Viable composition of the fecal microbiota and of the freeze-dried powder used for hFMT evaluated through a standard microbiology cultivation approach. Missing bar-charts refer to a microorganism below the detection limit. (EPS 625 kb)

**Additional file 6: Figure S5**. Supplementary results for the time-series analysis. (A) Chao1 alpha-diversity indices at baseline, Wilcoxon-Mann test with BH correction: ***P < 0.001. (n mice/treatment at baseline: control = 20, hFMT = 19, Lab4 = 20, vancomycin = 19). (B) Heatmap of the differentially abundant genera amongst treatments at baseline. Median abundances were scaled according to the row Z-score. Only genera with *P* < 0.05 are represented. (C) Alpha-diversity indices between timepoints in each treatment group (n mice per treatment/timepoint: control baseline = 16, mid = 20; hFMT baseline = 15, mid = 24; Lab4 baseline = 14, mid = 22; vancomycin baseline = 20, mid = 28), irrespective of the immunizations. Wilcoxon-Mann test with BH correction: ***P < 0.005; **P < 0.01; *P < 0.05.

**Additional file 7: Figure S6**. Correlation analysis between bacterial biomarkers and disease features. Pearson’s correlation coefficient (r) was used to test the correlations between bacterial biomarkers associated to both treatments and immunizations (from Fig. 1e and g) in (A) ddH2O, (B) vancomycin, (C) Lab4 and (D) hFMT treatment group at the final timepoint. Only TSHR immunization is shown. Only correlations with P < 0.05 are shown and the strength of the correlation is represented by the change in color from blue (negative) to red (positive correlation). (EPS 262 kb)

**Additional file 8: Figure S7**. Mice eye signs and orbital tissues abnormalities analysed histologically. (A) Mice eye signs indicating orbital disease. Representative images of a ßgal mouse lacking pathological eye signs and of a TSHR-immunized mouse with acute signs of inflammation and/or proptosis. (B) UCP-1 (uncoupling protein -1) as a marker for brown fat tissue (BAT). Elevated portions of small vacuoled BAT were present in TSHR-immunized mice. Representative pictures of stainings are shown. (C, D) CD3 as a marker for T cells. Some CD3^+^ T cells (indicated by arrows) were detected in adipose tissues (C) and in muscle tissues (D) of βgal and TSHR-immunized mice. Immunohistochemistry of orbital tissues was carried out as described in detail before [40]

**Additional file 9: Figure S8**. Caging effects during the hFMT engraftment did not impact the induced phenotype. (A) Non-metric dimensional scaling (NMDS) based on Bray-Curtis distances according to cages, specifically for the hFMT-receiving mouse group in each of the timepoints sampled. Baseline and mid-timepoint used fecal samples, whilst the final timepoint was on LGI microbiota. (B) Firmicutes to Bacteroidetes ratio in each hFMT-cage and in each timepoint. Pairwise Wilcoxon-Mann test: **P < 0.05; ****P < 0.001, ns *P* > 0.05. (C and D) Re-analysis without the samples from cage 8, as considered to be “high-colonized mice”. (C) *Bacteroides* spp. relative abundances between immunizations and in each timepoint. Wilcoxon-Mann test: ns *P* > 0.05, **P < 0.01. (D) Random Forest confusion matrix of the classification for treatments (all immunizations together) using the endpoint LGI genus-level microbiota, as in Fig. 1e but without samples from cage 8. Diagonal boxes represent the number of samples correctly predicted. (n mice/group endpoint: control = 20, hFMT = 15, Lab4 = 20, vancomycin = 19). (E and F) Evaluation of the autoimmune hyperthyroidism (E) and the GO (F) Z-score with specific regard to the cage 8 samples. No significant differences were observed between other samples. (EPS 272 kb)

**Additional file 10: Figure S9**. Correlation between weight gain and clinical features. Pearson’s correlation coefficient (r) was used to test the correlations between weight and T4 (A) and TSAb (B). (EPS 11601 kb)

**Additional file 11: Table S2**. Number of positive mice for each disease parameter. Numbers of TSHR-immunized mice positive for thyroid-stimulating antibodies (TSAbs), increased circulating thyroxine (T4), hyperplastic thyroid morphology, orbital brown fat (BAT) enlargement or muscle fiber atrophy are reported. The threshold for positivity was defined as upper 99% CI of the corresponding βgal groups. **Table S3**. Classification of total outcome of autoimmune hyperthyroidism and orbitopathy. Disease classification was done along the Z-Score values in Figure 8. The number of mice is given in %. Subclinical disease (Z-Score <0): these mice displayed no overt signs of autoimmune hyperthyroidism or orbitopathy although they developed TSHR antibodies. Clinical disease (Z-Score >0): These mice displayed clear signs of autoimmune hyperthyroidism and/or orbital pathology. Clinical disease is classified in mild and moderate/severe in accordance with the Z Score values as indicated (mild: Z Score 01; moderate/severe: Z-Score >1).

**Additional file 12: Figure S10**. Schematic model. Modulation of the gut microbiota in a mouse model of Graves‘ orbitopathy has an impact on induced disease. Female BALB/c mice were immunized with TSHR-A subunit and their intestinal microbiota were depleted with antibiotics (vancomycin) or skewed with probiotics (Lab4) and human fecal material transfer (hFMT) from severely affected patients with known increased Firmicutes/Bacteroidetes ratio, in order to study the effects of the microbiome on induced Graves’ Disease (GD)/ Graves’ orbitopathy (GO). Incidence and severity of GD (TSHR autoantibodies, thyroid histology, thyroxine level) and GO (orbital fat and muscle histology), lymphocyte phenotype, cytokine profile and gut microbiota were analysed at sacrifice (~22 weeks) . The results show that, whilst microbiome manipulation with all treatments specifically alter microbiome composition, hFMT increased severity of GD-like disease but treatment with Lab4 exacerbated induced autoimmune hyperthyroidism and GO. Vancomycin led to a significant increase of the genus Bacteroides and less pronounced GD- and GO-like changes.

## Data Availability

16S rRNA gene sequencing reads generated in this work were submitted under the NCBI accession ID PRJNA635258.

## Abbreviations

ATD: Autoimmune thyroid disease
BAT: Brown adipose tissue
BH: Benjamini-Hochberg
CD: Cluster of differentiation
CHO: Chinese hamster ovary cells
CSS: Cumulative sum scaling
ddH_2_O: Deionized water
EAE: Experimental autoimmune encephalomyelitis
EAT: Experimental autoimmune thyroiditis
GALT: Gut-associated lymphoid tissue
GD: Graves’ disease
GO: Graves’ orbitopathy
hFMT: Humanized fecal material transplant
LGI: Lower gastrointestinal tract
MRD: Maximum recovery diluent
MRS: Man, Rogosa and Sharpe broth
MS: Multiple sclerosis
NMDS: Non-metric dimensional scaling
NOD: Non-obese diabetic
OTU: Operational taxonomic unit
PERMANOVA: Permutational analysis of variance
QIIME: Quantitative insights into microbial ecology
RF: Random forest
rRNA: Ribosomal RNA
S: Svedberg
SPF: Specific pathogen free
spp: Subspecies
T1D: Type-1 diabetes
T3: Triiodothyronine
T4: Thyroxine
Th: T helper cells
TRAb: Total TSHR antibodies
Tregs: Regulatory T cells
TSAbs: Thyroid-stimulating antibodies
TSH: Thyroid-stimulating hormone
TSHR: Thyroid-stimulating hormone receptor
βgal: Beta-galactosidase
