# Supplementary material for: Modulating gut microbiota in a mouse model of Graves’ orbitopathy and its impact on induced disease
Source: Microbiome. 2021 Feb 16;9:45. doi: 10.1186/s40168-020-00952-4 (PMC7888139; doi:10.1186/s40168-020-00952-4)
Supplement: Supplementary file 2 — Additional file 1: Supplementary methods. Table S1. Characteristics of patients with sight-threatening GO recruited at the University Hospital Duisburg-Essen providing samples for hFMT production. [file 40168_2020_952_MOESM2_ESM.docx]

**Table S1**: Characteristics of patients with sight-threatening GO recruited at the University Hospital Duisburg-Essen providing samples for hFMT production.

| Patient ID | Age | Gender | Smoking | TSH (mU/L) | fT4 (pmol/L) | TRAB (U/L) | orbital decompres-  sion | steroids and selenium |
| --- | --- | --- | --- | --- | --- | --- | --- | --- |
| GO1 | 40 | female | current | 2.72 | 13.2 | 1.45 | 2 | b |
| GO2 | 56 | male | never | 0.01 | 10.7 | 3.89 | 0 | b |
| GO3 | 57 | female | current | 5.76 | 14.2 | 14.75 | 1 | b |
| GO4 | 47 | male | current | 0.02 | 25.1 | n.a. | 2 | b/a |
| GO5 | 71 | female | current | 1.52 | 20.6 | 16.83 | 1 | b/a |
| GO6 | 48 | female | current | 5.2 | 16.1 | n.a. | 2 | b |

n.a: not assessed. b/a: before and after. orbital decompression: 0 no decompression, 1, one eye; 2, both eyes. Steroids and selenium: b, before surgery; b/a, before and after surgery.

**Additional methods**

*Media, culture conditions and dilutions for cultivating bacteria*

Horse blood agar (Horse blood agar base No.2 (CM0271) with added Defibrinated Horse Blood (SR0050); both Oxoid, Basingstoke, United Kingdom) was incubated under aerobic conditions for 24 hours at 36 +/- 1 °C. Dilutions from 10-2 to 10-7 were made and total cell count was measured. MacConkey agar No. 3 (CM0115, Oxoid, Basingstoke, United Kingdom) was incubated under aerobic conditions for 24 hours at 36 +/- 1 °C. Dilutions from 10-2 to 10-7 were made and Enterobacteriaceae (red colonies with bile precipitation and straw colored colonies) were counted. Slanetz and Bartley Medium (CM0377, Oxoid, Basingstoke, United Kingdom) was incubated under aerobic conditions for 24 hours at 36 +/- 1 °C. Dilutions from 10^-2^ to 10^-7^  were prepared and enterococci (deep red colored colonies) were counted. Brilliance *E. coli* / coliform selective agar (CM1046, Oxoid, Basingstoke, United Kingdom) was incubated under aerobic conditions for 24 hours at 36 +/- 1 °C. Dilutions from 10^-2^ to 10^-7^ were made and *E. coli* (purple colonies) were counted. Tergitol 7 agar (CM0793, Oxoid, Basingstoke, United Kingdom) was incubated under aerobic conditions for 24 hours at 36 +/- 1 °C. Dilutions from 10-2 to 10-7 were prepared and coliforms (any color) were counted. Baird Parker agar base (CM0275, Oxoid, Basingstoke, United Kingdom) with added 50 ml of Egg Yolk Tellurite Emulsion (SR0054, Oxoid, Basingstoke, United Kingdom) was incubated under aerobic conditions for 48 hours at 36 +/- 1 °C. Dilutions from 10-2 to 10-7 were made and *Staphylococcus aureus* (black, shiny colonies with white and clear zones) were counted. Anaerobe basal agar (CM0972, Oxoid, Basingstoke, United Kingdom) was pre-reduced and incubated under anaerobic conditions for 48 hours at 36 +/- 1 °C. Dilutions from 10-2 to 10-7 were prepared and total cell count was measured. Each colony was checked for aerobic growth and ignored if so. Dichloran Rose-Bengal Chloramphenicol Agar (DRBC agar) (CM0727, Oxoid, Basingstoke, United Kingdom) was incubated under aerobic conditions for 48 hours at 36 +/- 1 °C. Dilutions from 10-1 to 10-2 were made and total yeast cell count was measured. Wilkins-Chalgren anaerobe agar (Code: CM0619, Oxoid, Basingstoke, United Kingdom) with added 1 vial of G-N Anaerobe Supplement (SR0108) and 25 ml defibrinated blood (SR0050/SR0051, both Oxoid, Basingstoke, United Kingdom) was pre-reduced and incubated under anaerobic conditions for 48 hours at 36 +/- 1 °C. Dilutions from 10-3 to 10-8 were made and *Bacteroides* spp. (grey/white colonies partially mucoid and with tattered edges) were counted. Each colony was checked for aerobic growth and ignored if so. MRS agar (CM0361, Oxoid, Basingstoke, United Kingdom) with added 1 vial of polymyxin B supplement (SR0099, Oxoid, Basingstoke, United Kingdom) was pre-reduced and incubated under anaerobic conditions for 48 hours at 36 +/- 1 °C. Dilutions from 10^-3^ to 10^-8^ were prepared and Lactobacilli (pale straw colored colonies) were measured. MRS-X agar (see MRS agar added 0.25 g L-cysteine hydrochloride monohydrate, 1 g Lithium chloride and 1.5 g Sodium propionate dissolved in 500 ml deionized water) was pre-reduced and incubated under anaerobic conditions for 48 hours at 36 +/- 1 °C. Dilutions from 10^-3^ to 10^-8^ were made and checked for bifidobacteria (small, shiny colonies). Alcohol shock anaerobe basal agar was prepared as follows: 1 ml of the 1 g faeces diluted in 9 ml maximum recovery diluent with glycerol was mixed with 1 ml ethanol (≥98%, Sigma Aldrich, St. Louis, Missouri, USA) and rolled for 30 min. Conditions were similar to anaerobe basal agar. Dilutions from 10-1 to 10-4 were made and total cell count was measured.  Each colony was checked for aerobic growth and ignored if so. Briefly, assessment of colony forming units (CFU) from total aerobe and total anaerobe bacteria as well as Enterobacteriaceae, enterococci, coliforms, staphylococci, yeast, *Bacteroides*, lactobacilli, bifidobacteria and clostridia was performed. In the case of growth on the agar plates, bacteria were identified by Gram staining, colony morphology, the presence of spores, catalase reaction and partially by the API system (BioMerieux, Marcy-l’Étoile, France). Viable bacterial cell counts were enumerated and all counts were recorded as the numbers of log10 colony forming units per gram of sample. Detection limits are the following according to the culture medium used: 1000 CFU/g feces for *Bacteroides*, 1000 CFU/g feces for bifidobacteria, 100 CFU/g feces for coliforms, 100 CFU/g feces for enterobacteria, 100 CFU/g feces for enterococci, 100 CFU/g feces for *E. coli*, 1000 CFU/g feces for lactobacilli, 100 CFU/g feces for staphylococci, 100 CFU/g feces for total aerobes, 1000 CFU/g feces for total anaerobes and 10 CFU/g feces for Yeast. Counts data were Box-Cox transformed before statistical analysis [[30]](https://paperpile.com/c/VLin6u/BzST).

*Detailed statistical methods*

Within each immunization group (TSHR or βgal), differences in bacterial relative abundances amongst treatments were tested using a linear regression model, correcting for the source of the anatomical site sampled (e.g. colon and entire). Pairwise differences between treatments were tested using a pairwise t-test with Benjamini-Hochberg (BH) adjustment for multiple corrections. Within each treatment (either ddH_2_O, Lab4, hFMT or vancomycin), differences between the two immunizations were assessed using a Welch’s t-test for unequal variance, with BH adjustment. Random Forest (RF) was employed to classify samples either amongst treatments (ddH_2_O, hFMT, Lab4 or vancomycin) or between immunizations (βgal or TSHR) based on their microbiota composition, and to identify genera driving the classification (variable importance). Relative abundance counts with non-zero values in at least 20% samples were retained, scaled and centred. To estimate the accuracy of prediction, a 10-fold cross-validation repeated 3 times (repeatedcv) method was used. The tuning hyperparameter *mtry*, calculated around the square root of the number of variables of the dataset, was tuned testing from 10 to 50 (step = 5), with 5,000 or 10,000 trees (ntree), using the R package Caret. RF was run using the identified parameter values providing the highest prediction accuracy during the cross-validation step using the R package 'RandomForest‘. The mean decrease Gini was used for the variable importance selection.

Disease features were grouped into specific categories such as: Lymph node T cells (CD4^+^CD25^+^ and CD4^+^ T cells), orbital pathology (muscular atrophy, brown fat and total fat), thyroid function and auto-antibodies (T4, TRAb and mTSAb). Finite values were correlated to the abundance of microbial biomarkers from the large intestine (obtained from the RF analysis) in each treatment and per immunization through the Pearson’s correlation coefficient (*r*), using the Corrplot R package.

The SourceTracker R package [[36]](https://paperpile.com/c/VLin6u/KTPo) was used to determine the possible transfer of taxonomies from donors to recipients – or engraftment [[37]](https://paperpile.com/c/VLin6u/9vGy) - as a result of the hFMT in mice. The GO patients and the ddH_2_O mice microbiota were used as “source” while the hFMT microbiota was used as “sink” (defined as "test“, figure 3E). To test the specificity of the hFMT engraftment, the hFMT and human microbiota communities were used as “source” and the murine ddH_2_O microbiota as “sink” (defined to as a control). The SourceTracker was run on the filtered OTU table at the family taxonomic level and default parameters (10 restart Gibbs sampling, 100 burn-in iterations for Gibbs sampling and 1,000 rarefaction depth). Counts that could not be assigned to a source at a certain significant threshold (a=0.001) were defined as “unknown”. Differences between % similarity to humans in test *vs.* control in each timepoint were tested with Fisher's exact test with Yates’ continuity correction. The test statistic was calculated considering only the number of observations >10% similarity to human source between analysis (test and control).
