# Supplementary figures and images for: Modulating gut microbiota in a mouse model of Graves’ orbitopathy and its impact on induced disease
Source: Microbiome. 2021 Feb 16;9:45. doi: 10.1186/s40168-020-00952-4 (PMC7888139; doi:10.1186/s40168-020-00952-4)

A

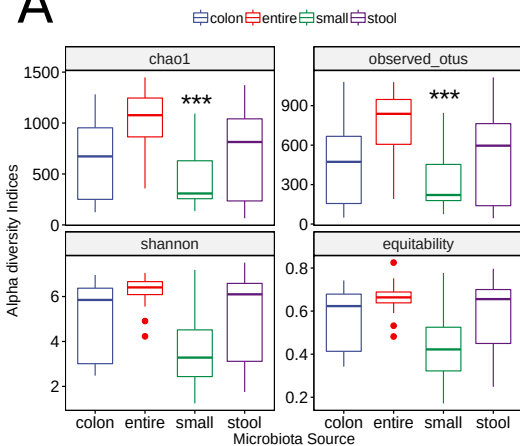

B

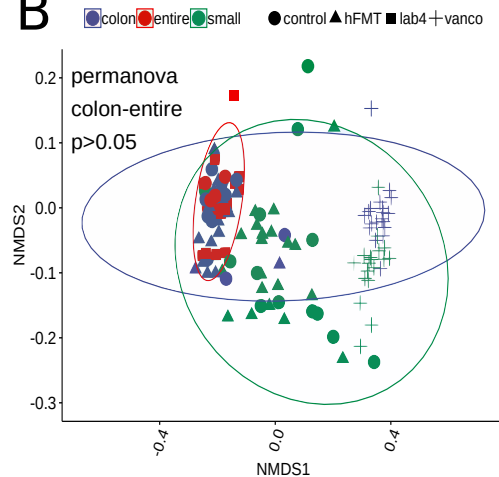

C

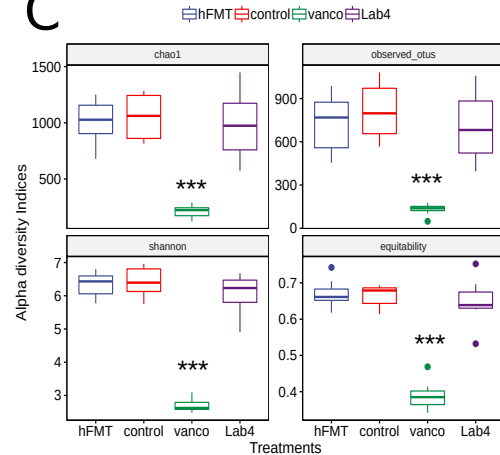

D

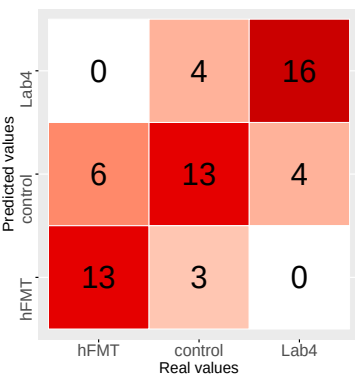

E

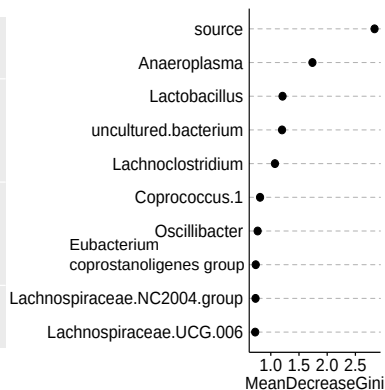

F

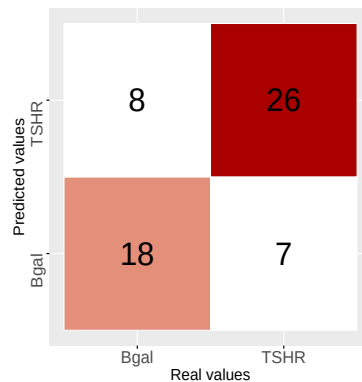

G

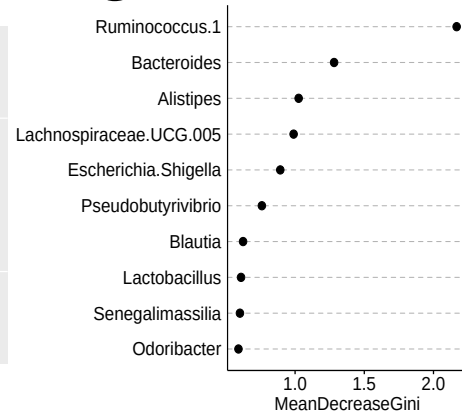

Supplement: Supplementary file 3 — Additional file 2: Figure S1. Supplementary results for the endpoint gut microbiota analysis. (A and B) Microbiota composition according to different anatomical samples in TSHR and βgal-immunized mice (samples/source: colon = 48, entire = 30, small = 51 from final timepoint and stool = 105 from baseline and mid-timepoint). (A) Alpha-diversity indices of the source of the microbiota sampled, Wilcoxon-Mann BH corrected test: ***P < 0.05. (B) NMDS of Bray-Curtis distances according to immunizations and sources at the endpoint. PERMANOVA between entire-colon samples P > 0.05. (C and D) Endpoint composition of the LGI microbiota amongst treatments in βgal-immunized mice. (n βgal mice/treatment at endpoint: control = 8, hFMT = 8, Lab4 = 10, vancomycin = 19). (C) Alpha diversity amongst treatments, Wilcoxon-Mann BH corrected test:***P < 0.001. (D to G) RandomForest of a model excluding vancomycin samples (n mice/treatment endpoint: control = 20, hFMT = 19, Lab4 = 20). (D) Confusion matrix for treatments w/o vancomycin samples. Diagonal boxes represent the number of samples correctly predicted. (E) Top-10 variables of treatment classification according to the Mean Decrease Gini, including the microbiota source as an effect related to figure E. (F) Confusion matrix for immunizations in a model w/o vancomycin samples. (n mice/immunization endpoint: TSHR = 33 and βgal = 26). (G) Top-10 variables of immunizations classification according to the Mean Decrease Gini, including the microbiota source and treatments as an effect related to figure G. Wilcoxon-Mann test with BH correction: ***P < 0.005; **P < 0.01; *P < 0.05. [file 40168_2020_952_MOESM3_ESM.pdf]

Figure S2: Differential abundant genera in TSHR-immunised mice

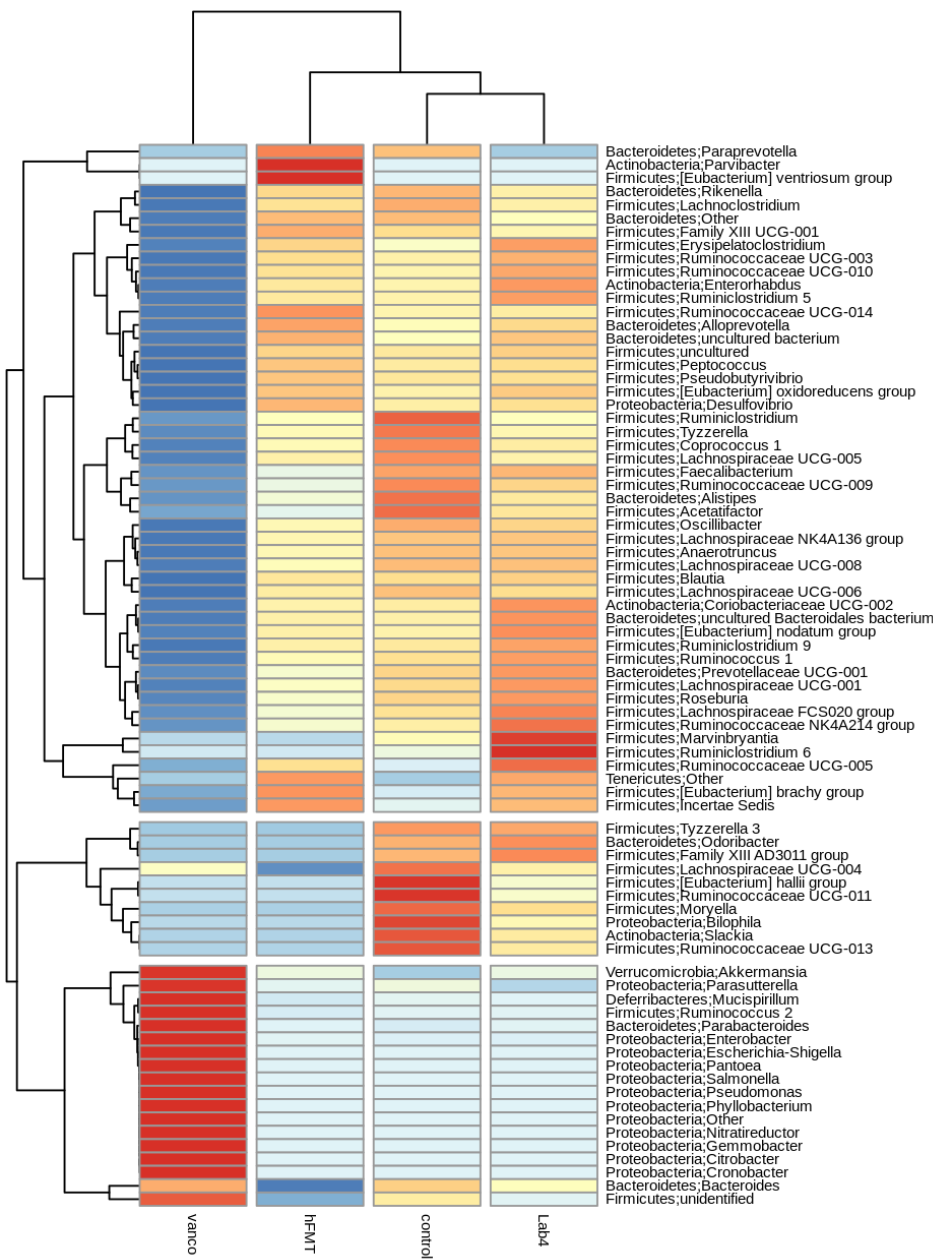

Supplement: Supplementary file 4 — Additional file 3: Figure S2. Heatmap of the differentially abundant genera amongst treatments in TSHR-immunized mice. Median abundances were scaled according to row Z-score. Only genera with P < 0.5 are represented. [file 40168_2020_952_MOESM4_ESM.pdf]

Figure S3: Differential abundant genera in Bgal mice

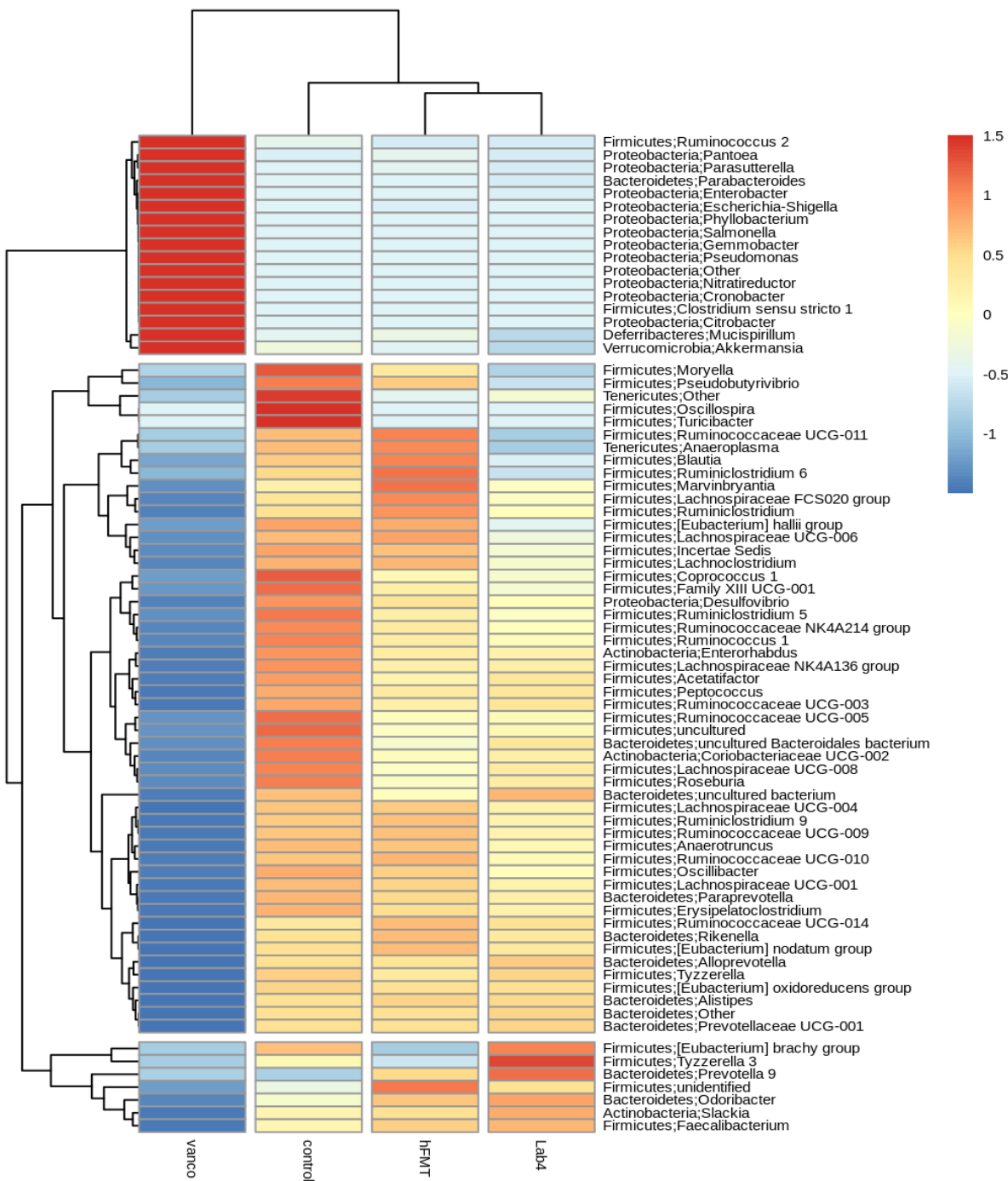

Supplement: Supplementary file 5 — Additional file 4: Figure S3. Heatmap of the differentially abundant genera amongst treatments in βgal mice. Median abundances were scaled according to row Z-score. Only genera with P < 0.5 are represented. [file 40168_2020_952_MOESM5_ESM.pdf]

## Treatments

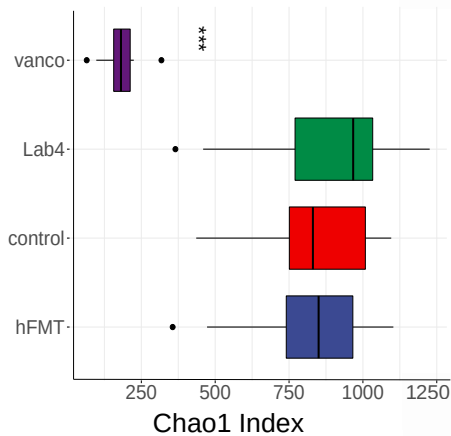

# B

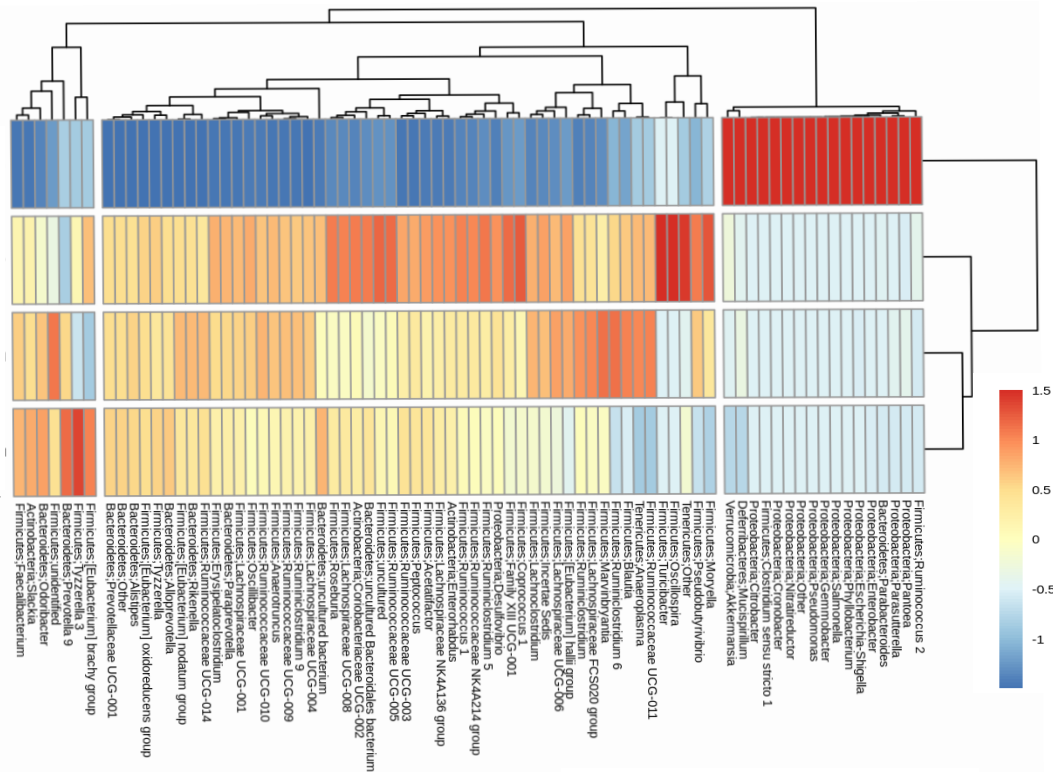

C

## Alpha diversity indices

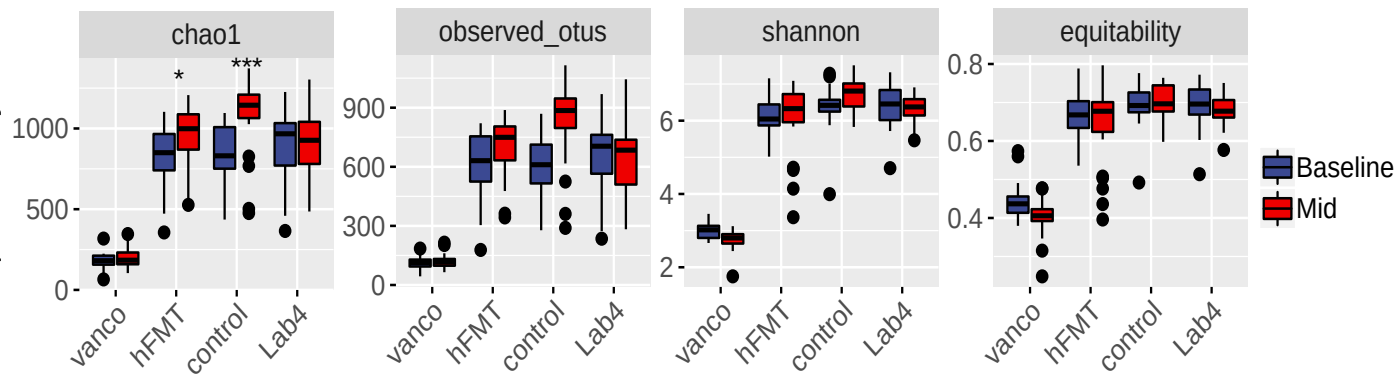

Supplement: Supplementary file 7 — Additional file 6: Figure S5. Supplementary results for the time-series analysis. (A) Chao1 alpha-diversity indices at baseline, Wilcoxon-Mann test with BH correction: ***P < 0.001. (n mice/treatment at baseline: control = 20, hFMT = 19, Lab4 = 20, vancomycin = 19). (B) Heatmap of the differentially abundant genera amongst treatments at baseline. Median abundances were scaled according to the row Z-score. Only genera with P < 0.05 are represented. (C) Alpha-diversity indices between timepoints in each treatment group (n mice per treatment/timepoint: control baseline = 16, mid = 20; hFMT baseline = 15, mid = 24; Lab4 baseline = 14, mid = 22; vancomycin baseline = 20, mid = 28), irrespective of the immunizations. Wilcoxon-Mann test with BH correction: ***P < 0.005; **P < 0.01; *P < 0.05. [file 40168_2020_952_MOESM7_ESM.pdf]

# Suppl. Figure S7

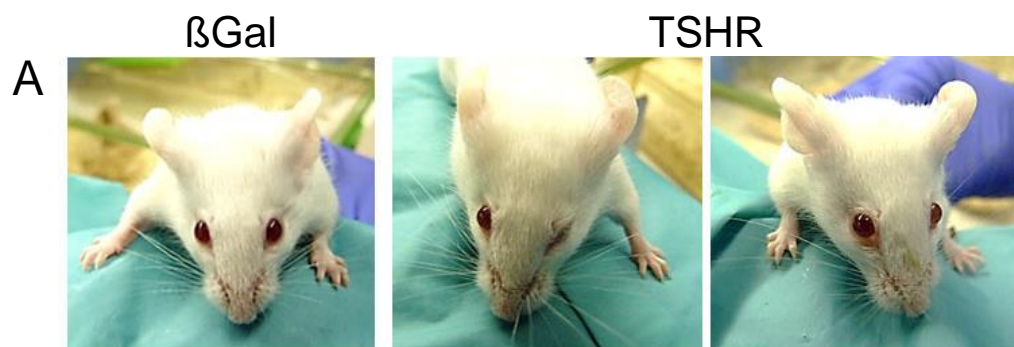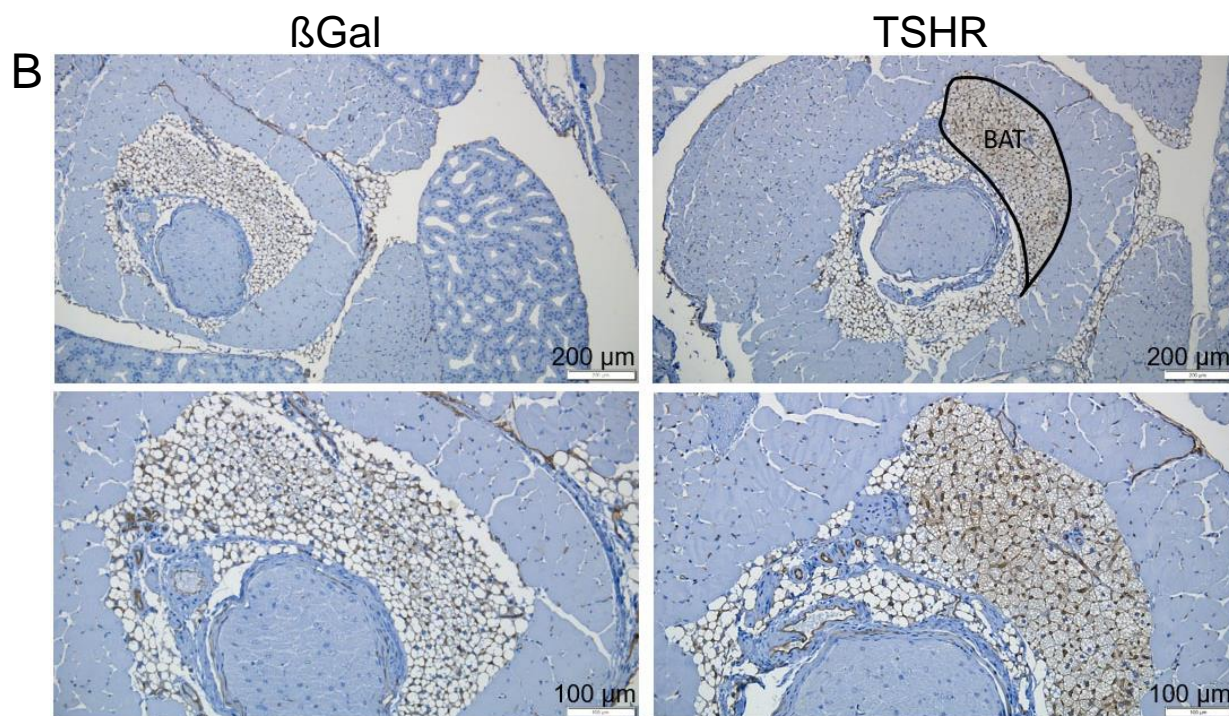

Suppl. Figure S7

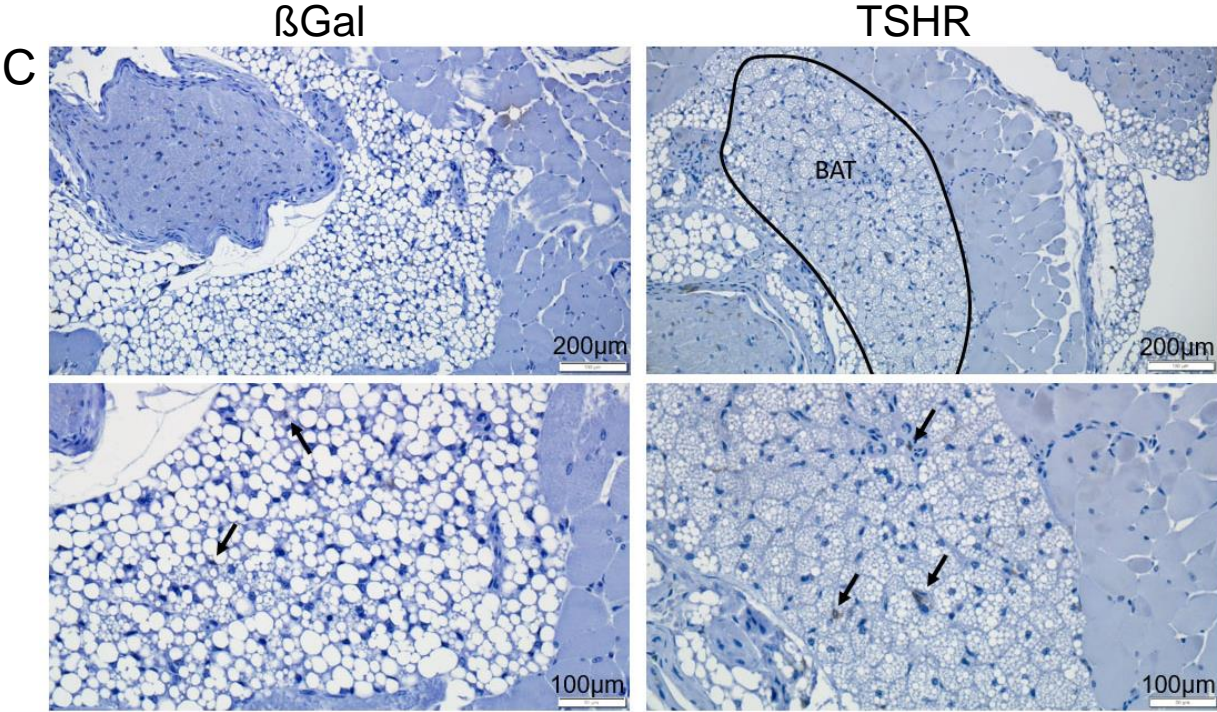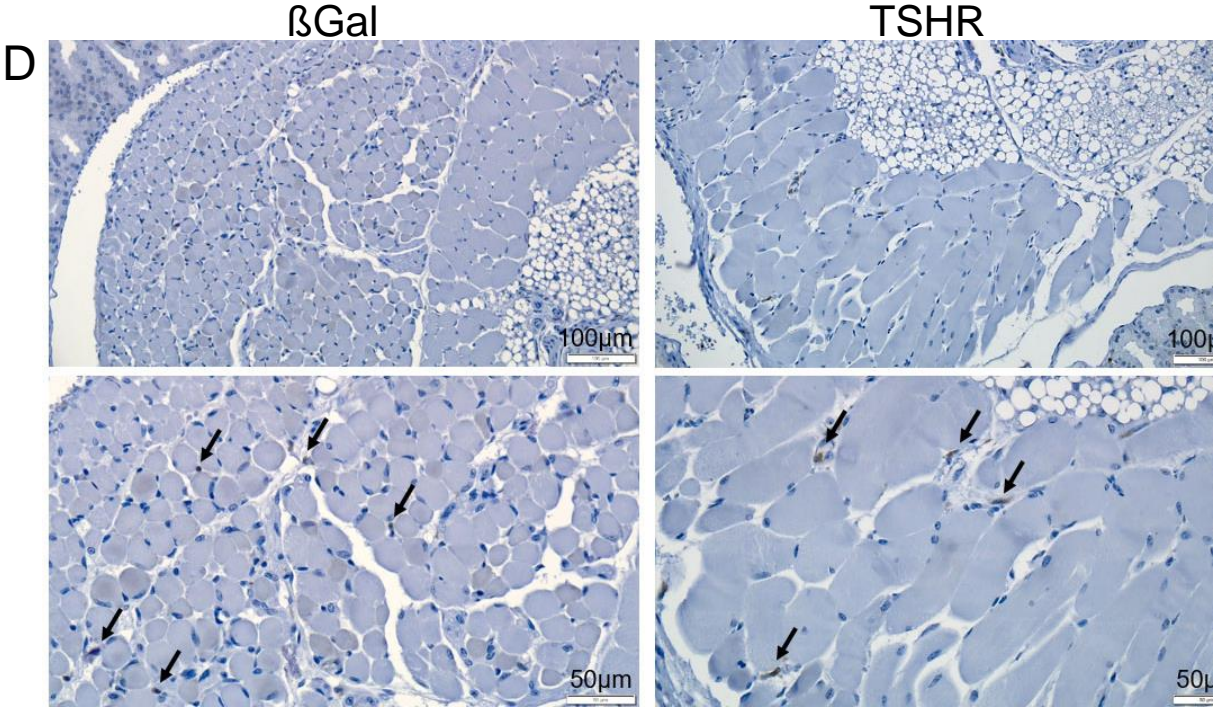

Supplement: Supplementary file 9 — Additional file 8: Figure S7. Mice eye signs and orbital tissues abnormalities analysed histologically. (A) Mice eye signs indicating orbital disease. Representative images of a ßgal mouse lacking pathological eye signs and of a TSHR-immunized mouse with acute signs of inflammation and/or proptosis. (B) UCP-1 (uncoupling protein -1) as a marker for brown fat tissue (BAT). Elevated portions of small vacuoled BAT were present in TSHR-immunized mice. Representative pictures of stainings are shown. (C, D) CD3 as a marker for T cells. Some CD3+ T cells (indicated by arrows) were detected in adipose tissues (C) and in muscle tissues (D) of βgal and TSHR-immunized mice. Immunohistochemistry of orbital tissues was carried out as described in detail before [40] [file 40168_2020_952_MOESM9_ESM.pdf]

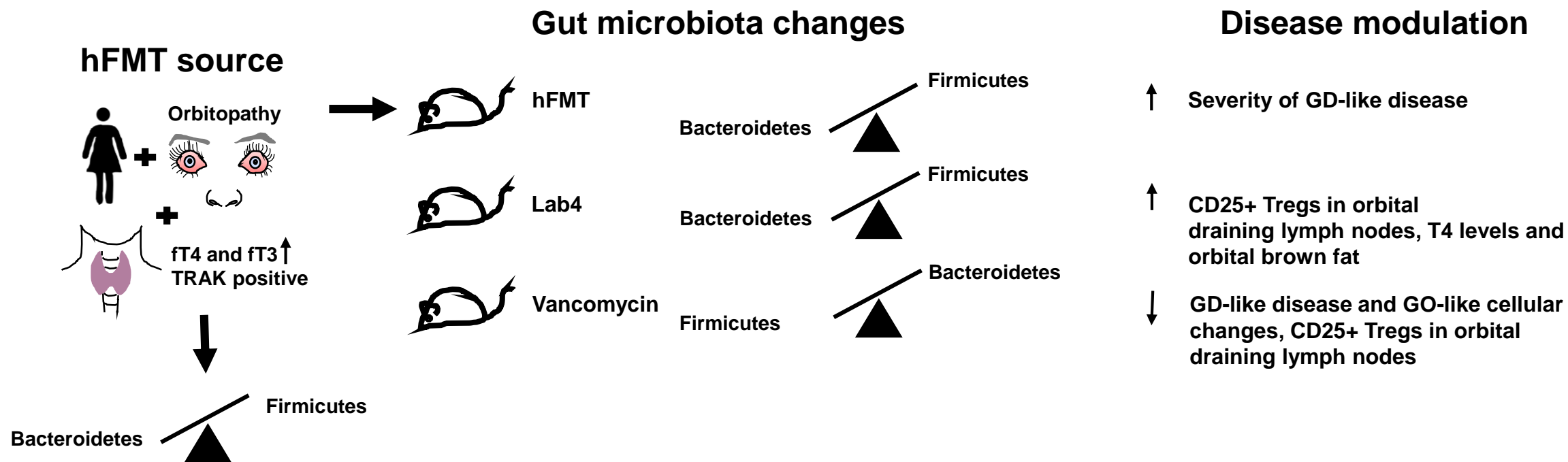

Supplement: Supplementary file 13 — Additional file 12: Figure S10. Schematic model. Modulation of the gut microbiota in a mouse model of Graves‘ orbitopathy has an impact on induced disease. Female BALB/c mice were immunized with TSHR-A subunit and their intestinal microbiota were depleted with antibiotics (vancomycin) or skewed with probiotics (Lab4) and human fecal material transfer (hFMT) from severely affected patients with known increased Firmicutes/Bacteroidetes ratio, in order to study the effects of the microbiome on induced Graves’ Disease (GD)/ Graves’ orbitopathy (GO). Incidence and severity of GD (TSHR autoantibodies, thyroid histology, thyroxine level) and GO (orbital fat and muscle histology), lymphocyte phenotype, cytokine profile and gut microbiota were analysed at sacrifice (~22 weeks) . The results show that, whilst microbiome manipulation with all treatments specifically alter microbiome composition, hFMT increased severity of GD-like disease but treatment with Lab4 exacerbated induced autoimmune hyperthyroidism and GO. Vancomycin led to a significant increase of the genus Bacteroides and less pronounced GD- and GO-like changes. [file 40168_2020_952_MOESM13_ESM.pdf]
