# Supplementary material for: Modulating gut microbiota in a mouse model of Graves’ orbitopathy and its impact on induced disease
Source: Microbiome. 2021 Feb 16;9:45. doi: 10.1186/s40168-020-00952-4 (PMC7888139; doi:10.1186/s40168-020-00952-4)
Supplement: Supplementary file 12 — Additional file 11: Table S2. Number of positive mice for each disease parameter. Numbers of TSHR-immunized mice positive for thyroid-stimulating antibodies (TSAbs), increased circulating thyroxine (T4), hyperplastic thyroid morphology, orbital brown fat (BAT) enlargement or muscle fiber atrophy are reported. The threshold for positivity was defined as upper 99% CI of the corresponding βgal groups. Table S3. Classification of total outcome of autoimmune hyperthyroidism and orbitopathy. Disease classification was done along the Z-Score values in Figure 8. The number of mice is given in %. Subclinical disease (Z-Score <0): these mice displayed no overt signs of autoimmune hyperthyroidism or orbitopathy although they developed TSHR antibodies. Clinical disease (Z-Score >0): These mice displayed clear signs of autoimmune hyperthyroidism and/or orbital pathology. Clinical disease is classified in mild and moderate/severe in accordance with the Z Score values as indicated (mild: Z Score 01; moderate/severe: Z-Score >1). [file 40168_2020_952_MOESM12_ESM.docx]

**Table S2**. Number of positive mice for each disease parameters

|  | TSAb | T4 | hyperplastic | BAT | Atrophy |
| --- | --- | --- | --- | --- | --- |
| ddH_2_O | 11/13  =84% | 8/12  =66.7% | 6/13  =46% | 9/13  =62.9% | 7/10  =70% |
| Lab4 | 10/11  =90.9% | 8/11  =73% | 6/10  =60% | 9/11  =81.8% | 7/11  =63.6% |
| hFMT | 12/12  =100% | 9/12  =75% | 10/12  =83% | 9/9  =100% | 4/9  =44.4% |
| Vancomycin | 9/14  =64.3% | 5/14  =35.7% | 5/13  =38% | 7/13  =53.8% | 8/12  =66.7% |

Numbers of TSHR-immunized mice positive for thyroid stimulating antibodies (TSAbs), increased circulating thyroxine (T4), hyperplastic thyroid morphology, orbital brown fat (BAT) enlargement or muscle fiber atrophy are reported. The threshold for positivity was defined as upper 99% CI of the corresponding βgal groups.

**Table S3**. Classification of total outcome of autoimmune hyperthyroidism and orbitopathy

| Disease classification | Z-score | ddH_2_O mice (%) | Lab4 mice  (%) | hFMT mice (%) | Vancomycin mice (%) |
| --- | --- | --- | --- | --- | --- |
| **Autoimmune hyperthyroidism** | | | | | |
| Subclinical | ≦0 | 28.6 | 18.2 | 18.2 | 50.0 |
| Clinical | >0 | 71.4 | 81.8 | 81.8 | 50.0 |
| *Mild* | 0≧1 | 35.7 | 9.1 | 54.8 | 40.9 |
| *Moderate-severe* | >1 | 35.7 | 72.7 | 27.3 | 7.1 |
| **Graves’ orbitopathy** | | | | | |
| Subclinical | ≦0 | 15.4 | 27.3 | 33.3 | 57.1 |
| Clinical | >1 | 84.6 | 72.7 | 66.7 | 42.9 |
| *Mild* | 0≧1 | 38.4 | 36.3 | 44.5 | 35.8 |
| *Moderate-severe* | >1 | 46.2 | 36.3 | 22.2 | 7.1 |

Disease classification was done along the Z-Score values in Figure 8. The number of mice is given in %. Subclinical disease (Z-Score <0): these mice displayed no overt signs of autoimmune hyperthyroidism or orbitopathy although they developed TSHR antibodies. Clinical disease (Z-Score >0): These mice displayed clear signs of autoimmune hyperthyroidism and/or orbital pathology. Clinical disease is classified in mild and moderate/severe in accordance with the Z Score values as indicated (mild: Z Score 01; moderate/severe: Z-Score >1).
